# Computational-experimental approach to drug-target interaction mapping: A case study on kinase inhibitors

**DOI:** 10.1371/journal.pcbi.1005678

**Published:** 2017-08-07

**Authors:** Anna Cichonska, Balaguru Ravikumar, Elina Parri, Sanna Timonen, Tapio Pahikkala, Antti Airola, Krister Wennerberg, Juho Rousu, Tero Aittokallio

**Affiliations:** 1 Helsinki Institute for Information Technology HIIT, Department of Computer Science, Aalto University, Espoo, Finland; 2 Institute for Molecular Medicine Finland FIMM, University of Helsinki, Helsinki, Finland; 3 Department of Information Technology, University of Turku, Turku, Finland; 4 Department of Mathematics and Statistics, University of Turku, Turku, Finland; Icahn School of Medicine at Mount Sinai, UNITED STATES

## Abstract

Due to relatively high costs and labor required for experimental profiling of the full target space of chemical compounds, various machine learning models have been proposed as cost-effective means to advance this process in terms of predicting the most potent compound-target interactions for subsequent verification. However, most of the model predictions lack direct experimental validation in the laboratory, making their practical benefits for drug discovery or repurposing applications largely unknown. Here, we therefore introduce and carefully test a systematic computational-experimental framework for the prediction and pre-clinical verification of drug-target interactions using a well-established kernel-based regression algorithm as the prediction model. To evaluate its performance, we first predicted unmeasured binding affinities in a large-scale kinase inhibitor profiling study, and then experimentally tested 100 compound-kinase pairs. The relatively high correlation of 0.77 (p < 0.0001) between the predicted and measured bioactivities supports the potential of the model for filling the experimental gaps in existing compound-target interaction maps. Further, we subjected the model to a more challenging task of predicting target interactions for such a new candidate drug compound that lacks prior binding profile information. As a specific case study, we used tivozanib, an investigational VEGF receptor inhibitor with currently unknown off-target profile. Among 7 kinases with high predicted affinity, we experimentally validated 4 new off-targets of tivozanib, namely the Src-family kinases *FRK* and *FYN A*, the non-receptor tyrosine kinase *ABL1*, and the serine/threonine kinase *SLK*. Our sub-sequent experimental validation protocol effectively avoids any possible information leakage between the training and validation data, and therefore enables rigorous model validation for practical applications. These results demonstrate that the kernel-based modeling approach offers practical benefits for probing novel insights into the mode of action of investigational compounds, and for the identification of new target selectivities for drug repurposing applications.

## Author summary

Significant efforts have been devoted in recent years to the development of machine learning models to support different stages of drug development process. Given the enormous size of the chemical universe, such models could offer a complementary and cost-effective means to experimental determination of drug-target interactions, toward prioritization of the most potent ones for further verification in the laboratory. In order to demonstrate the benefits of the prediction models in practical application cases, we carefully evaluated the predictive power of a well-established machine learning model in filling the gaps in existing profiling studies and prediction of target interactions for a new drug candidate. As a specific case study, we focused on kinase inhibitors, which form the largest class of new drugs approved for cancer treatment, but are also known to have wide multi-target activities contributing both to their therapeutic and toxic responses. The high agreement observed between the predicted and experimentally-measured drug-target bioactivities under the implemented rigorous validation setup demonstrates the potential of the machine learning approach, not only for filling the gaps in existing drug-target interaction maps, but also toward off-target interaction prediction for investigational drugs, and finding potential new uses for already approved drugs (drug repurposing).

## Introduction

Deregulated kinase activity plays a role in many diseases, hence calling for therapeutic compounds that could effectively inhibit specific members of the protein kinome. Although kinase inhibitors form the largest group of new drugs approved for cancer treatment [1], a majority of them are ATP-competitive, and therefore present a highly promiscuous mechanism of action (MoA), due to the high evolutionary conservation of the kinase ATP-binding pockets [2,3]. The polypharmacological interactions contribute both to therapeutic and toxic responses seen in clinically-approved and investigational kinase inhibitors. Thus, improved knowledge of the complex compound-target binding interactions across the full protein kinome, including both on- and off-target effects, is of high clinical relevance for future drug discovery applications.

Recent technological advances in chemoproteomic approaches, such as thermal profiling [4], have enabled efficient determination of kinome-wide compound potency. Several commercial providers are available for preclinical kinase inhibitor testing *in vitro*, including DiscoverX, Millipore and Reaction Biology. Even though the experimental compound-target interaction mapping is critical to characterizing a compound’s MoA, computational methods provide a complementary and cost-effective approach with the potential to accelerate the exploration of the enormous size of the chemical universe, estimated to consist of approximately 10^20^ molecules exhibiting good pharmacological properties [5]. The hypothesis is that *in silico* models could provide fast, large-scale and systematic pre-screening of chemical probes, toward prioritization of the most potent interactions for further *in vitro* or *ex vivo* verification in the laboratory [6–10].

In particular, a lot of work has been devoted to compound-based interaction prediction methods, including quantitative structure-activity relationship (QSAR) models, which aim to relate structural properties of the chemical molecules to their bioactivity profiles [11,12]. Another class of machine learning methods, so called target-based methods, focus on evaluating similarities between amino acid sequences or three-dimensional structures of protein targets [13]. In these supervised learning approaches, models are trained using available bioactivity data, together with either compound or protein information, which allows then predicting either new target interactions for a given drug or new drugs targeting a given protein. Furthermore, such methods typically focus on a limited set of molecules of interest.

As a more recent class of computational modelling approaches, systems-based frameworks take advantage of the information available on both compounds and targets. For instance, Yamanishi *et al*. proposed a supervised machine learning approach for categorizing drug-target pairs as interacting or non-interacting based on an integrated model of chemical and genomic molecular profiles [14]. Since this seminal work, a wide variety of systems-based prediction methods have been developed that utilize various molecular descriptors and learning techniques, including random forest, neural networks and kernel learning [15–32]. Even though such models may hold a great potential, their computational predictions are rarely being directly verified in the laboratory and, consequently, their practical benefits for the drug discovery or repurposing applications remain largely unknown.

**Fig 1:**
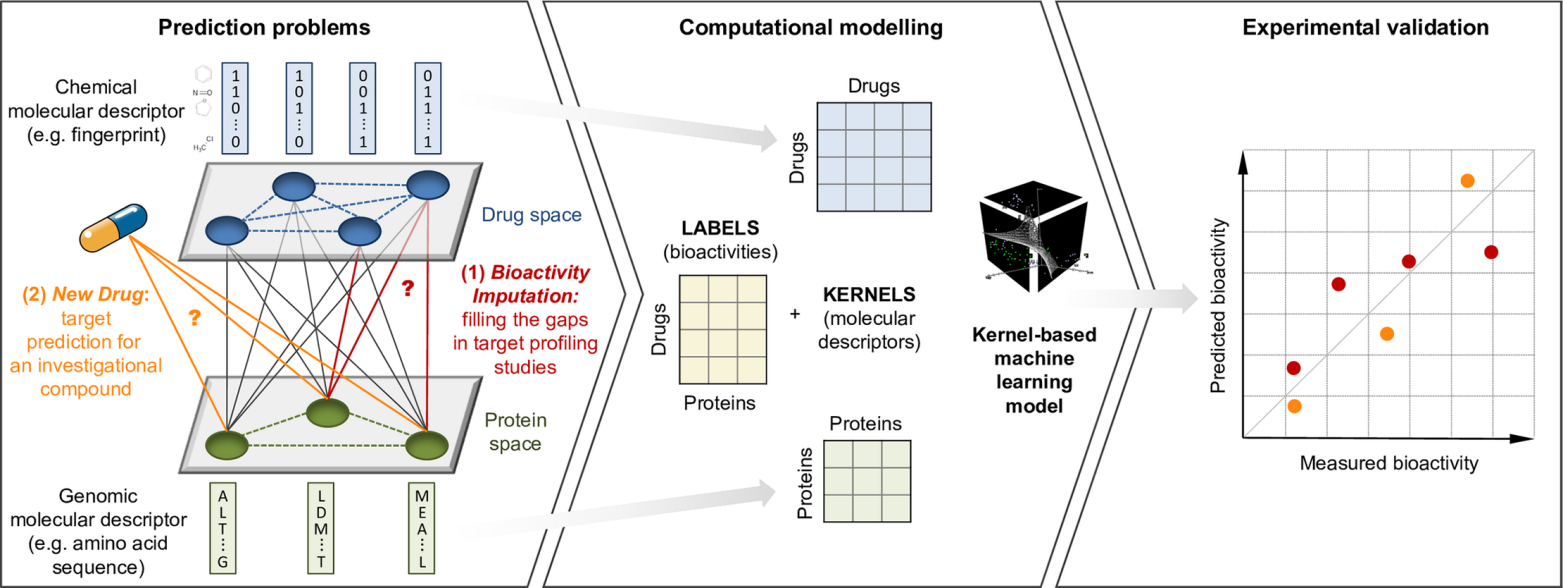
An overview of our computational-experimental framework for prediction and pre-clinical testing of compound-protein bioactivity profiles. Two separate prediction problems are considered: (1) filling the gaps in existing compound-target interaction maps and (2) prediction of target interactions for a new or investigational compound. Molecular descriptors of drug compounds and protein targets are encoded as kernels, and used for binding affinity prediction with a regularized least squares regression model KronRLS. Finally, a subset of predicted compound-protein bioactivities is experimentally tested (see Materials and Methods for details). Since the experimental validations do not exists at the time of making the predictions, this approach effectively assesses any potential model overfitting to the training data only. We chose to use kernel-based models as these are well-suited for representing structured objects, such as molecules, that cannot be accurately described by a standard feature vector. Different types of drug and protein kernels can be calculated using readily available chemical structures and amino acid sequences. The resulting matrices associate all pairs of input objects, and therefore a kernel function can be considered as a similarity measure.

Toward testing the practical potential of systems-based machine learning models, we implemented a computational-experimental framework for prediction and verification of compound-target bioactivity profiles (Fig 1). We focused on a regression problem, where the task is to predict the actual binding affinities, instead of the standard bioactivity classification setting that treats molecular interactions as simple on/off relationships. As a prediction model, we applied a well-established kernel-based regularized least squares learning algorithm (KronRLS [33]), because kernels, in addition to offering a computationally efficient means for increasing the power of linear learning algorithms, are particularly well-suited for capturing and learning complex molecular properties for prediction purposes [34,35].

The specific contributions of the work are the following. First, we evaluated a large number of molecular descriptors in the form of kernels, including our novel, extended target profile-based protein kernel, and a generic string kernel that has not previously been used in the context of compound-protein interaction prediction. Second, we show how these kernels guided us in filling the gaps in a large-scale compound-kinase interaction map [3]. Third, we experimentally tested a subset of 100 predicted binding affinities, achieving a high correlation of 0.77 between the measured and predicted bioactivities. Finally, we demonstrate the potential of the modelling approach in a more challenging task of predicting target selectivities for such a new candidate compound that has no bioactivity data available for model training. As a specific case study, we used an investigational tyrosine kinase inhibitor tivozanib whose established target profile consists only of 3 on-targets. We experimentally tested and validated 4 out of 7 kinases predicted as tivozanib’s off-targets, providing novel insights into its MoA, and thereby extending the potential therapeutic target space of tivozanib.

## Results

**Fig 2:**
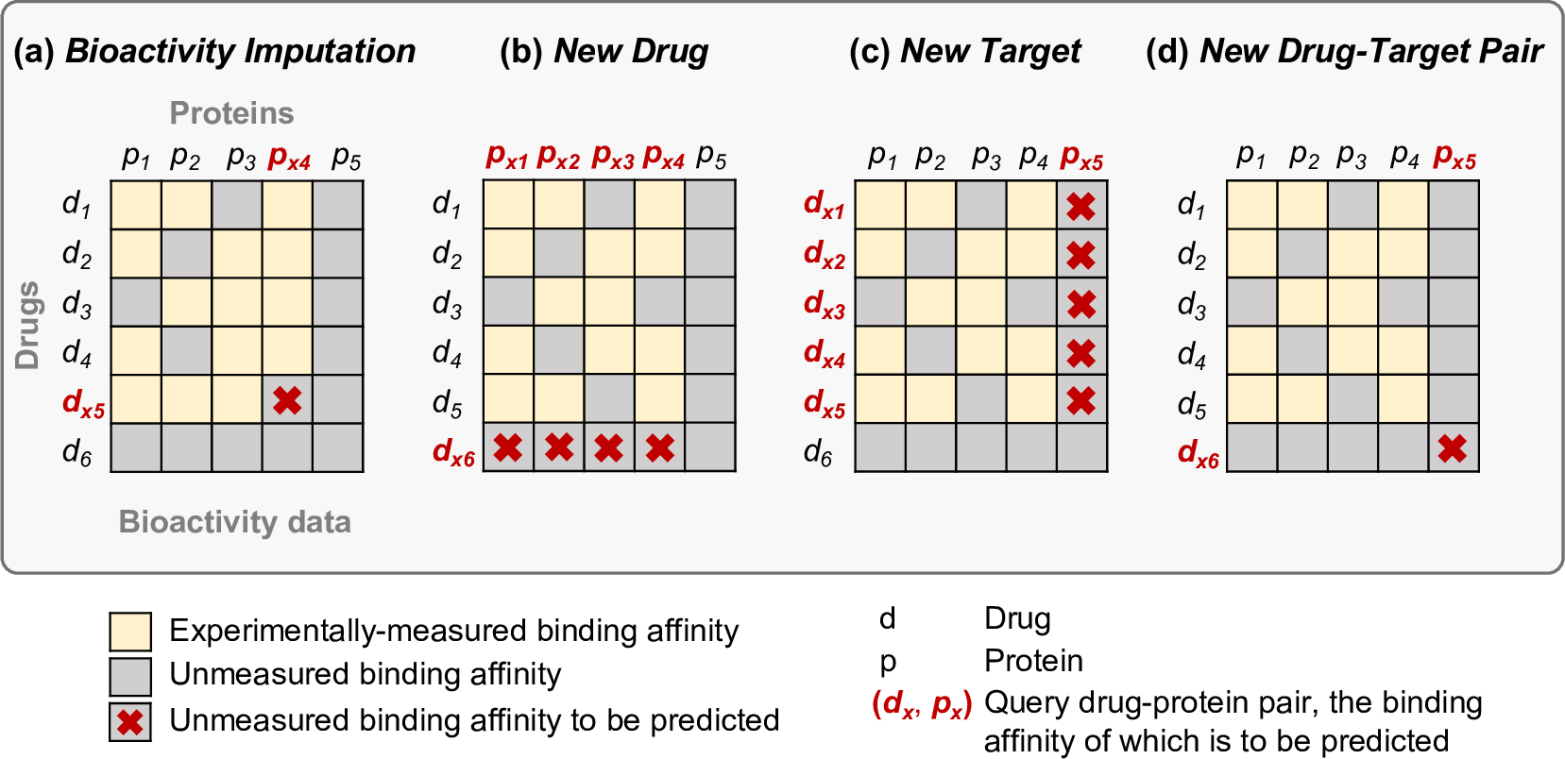
Drug-protein interaction prediction scenarios. (*d*_*x*_, *p*_*x*_) denotes a query drug-protein pair, the binding affinity of which is to be predicted. (**a**) The *Bioactivity Imputation* scenario: both the drug *d*_*x*_ and protein *p*_*x*_ are present in the training set, i.e., there exist known bioactivity values for the drug *d*_*x*_ and protein *p*_*x*_, but not for their interaction (*d*_*x*_, *p*_*x*_). (**b**) The *New Drug* scenario: the protein *p*_*x*_ is present in the training set, whereas the drug *d*_*x*_ is not, i.e., there exist known bioactivity values for the protein *p*_*x*_ but not for the drug *d*_*x*_. (**c**) The *New Target* scenario: the drug *d*_*x*_ is present in the training set, whereas the protein *p*_*x*_ is not, i.e., there exist known bioactivity values for the drug *d*_*x*_, but not for the protein *p*_*x*_. (**d**) The *New Drug-Target Pair* scenario: neither the drug *d*_*x*_ nor protein *p*_*x*_ is present in the training set, i.e., there exist no bioactivity values neither for the drug *d*_*x*_ nor protein *p*_*x*_. In this work, we focused primarily on two most common and practical prediction scenarios of (a) and (b), which correspond to filling the gaps in existing experimentally-measured drug-target interaction maps and prediction of target interactions for an investigational drug compound, respectively.

In the real use cases, the problem of compound-protein interaction prediction should be considered separately under four different scenarios, depending on whether or not the training and test sets share common compounds, proteins, or both (Fig 2 and S1 Table). In the below results sections, we focused on the two most common and practical scenarios of (1) filling the experimental gaps in compound-target profiling datasets (referred to as the *Bioactivity Imputation* scenario, Fig 2A) and (2) prediction of target interactions for an investigational drug compound (referred to as the *New Drug* scenario, Fig 2B). Moreover, S7 Fig shows, for comparison, the results obtained under the symmetric *New Target* scenario (Fig 2C).

### Evaluation of molecular descriptors for compound-target interaction inference

A key assumption in the systems-based compound-target interaction prediction algorithm is that similar drug compounds are likely to bind to similar protein targets, and therefore the first challenge lies in the representation and use of molecular similarities in the most predictive way. We encoded here similarities between drugs and similarities between proteins using different types of kernels, constructed based on chemical two- and three-dimensional structures, amino acid sequences, protein structures, and molecular interaction profiles (see Materials and Methods for details). Such systematic construction of chemical and genomic molecular descriptors resulted in 12 drug kernels and 8 protein kernels. To predict compound-protein binding affinities using the regression setup, we applied a regularized least squares (RLS) model for each pair of drug kernel and protein kernel (KronRLS algorithm, see Materials and Methods).

For computational evaluation of the predictive performance of various molecular descriptors and optimization of model parameters under separate prediction scenarios, we carried out two systematic nested cross-validation (CV) procedures, i.e., leave-one-out cross-validation (LOO-CV, S12 Fig) and leave-drug-out cross-validation (LDO-CV, S13 Fig), using 16,265 known binding affinities (pK_i_ values) between 152 kinase inhibitors and 138 protein kinases measured in a large-scale functional bioassay by Metz *et al*. [3] (S9 Fig). We applied LOO-CV to tune model parameters and to evaluate its predictive performance when filling experimental gaps in large-scale target profiling studies (the *Bioactivity Imputation* scenario, Fig 2A), and LDO-CV in the inference of target interactions for a new candidate drug compound *(*the *New Drug* scenario, Fig 2B*)*. LOO-CV corresponds to the design where scattered missing values are present in otherwise known compound-protein bioactivity matrix, and the aim is to predict the missing entries within the training data. LDO-CV, on the other hand, simulates more challenging inference problem, in which the aim is to predict targets of an investigational drug compound, not encountered in the training data (Materials and Methods, Tables 1 and S1).

**Table 1:** Applied nested cross-validation strategies.

	LOO-CV	LDO-CV^*a*^
**Aim**	To evaluate the performance of the prediction model in the task of filling the experimental gaps in existing compound-protein interaction maps (the *Bioactivity Imputation scenario*, Fig 2A).	To evaluate the performance of the prediction model in the task of predicting target interactions for a new candidate drug compound (the *New Drug scenario*, Fig 2B).
**Outer** **CV**	One entry (compound-protein pair) at a time is removed from the compound-protein interaction matrix **Y**, and kept as a test fold.	One row (compound) at a time is removed from the compound-protein interaction matrix **Y**, and kept as a test fold.
**Inner CV**	One entry (compound-protein pair) at a time is removed from the remaining part of the compound-protein interaction matrix **Y**, and kept as a test fold.	Five rows (compounds) at a time are removed at random from the remaining part of the compound-protein interaction matrix **Y**, and kept as a test fold.

^*a*^ In case of LDO-CV, the rows and columns corresponding to the compounds included in the test fold are removed from the drug kernel matrix **K**_*D*_ before model training.

#### *Bioactivity Imputation*: Computational evaluation of filling in the experimental gaps

**Fig 3:**
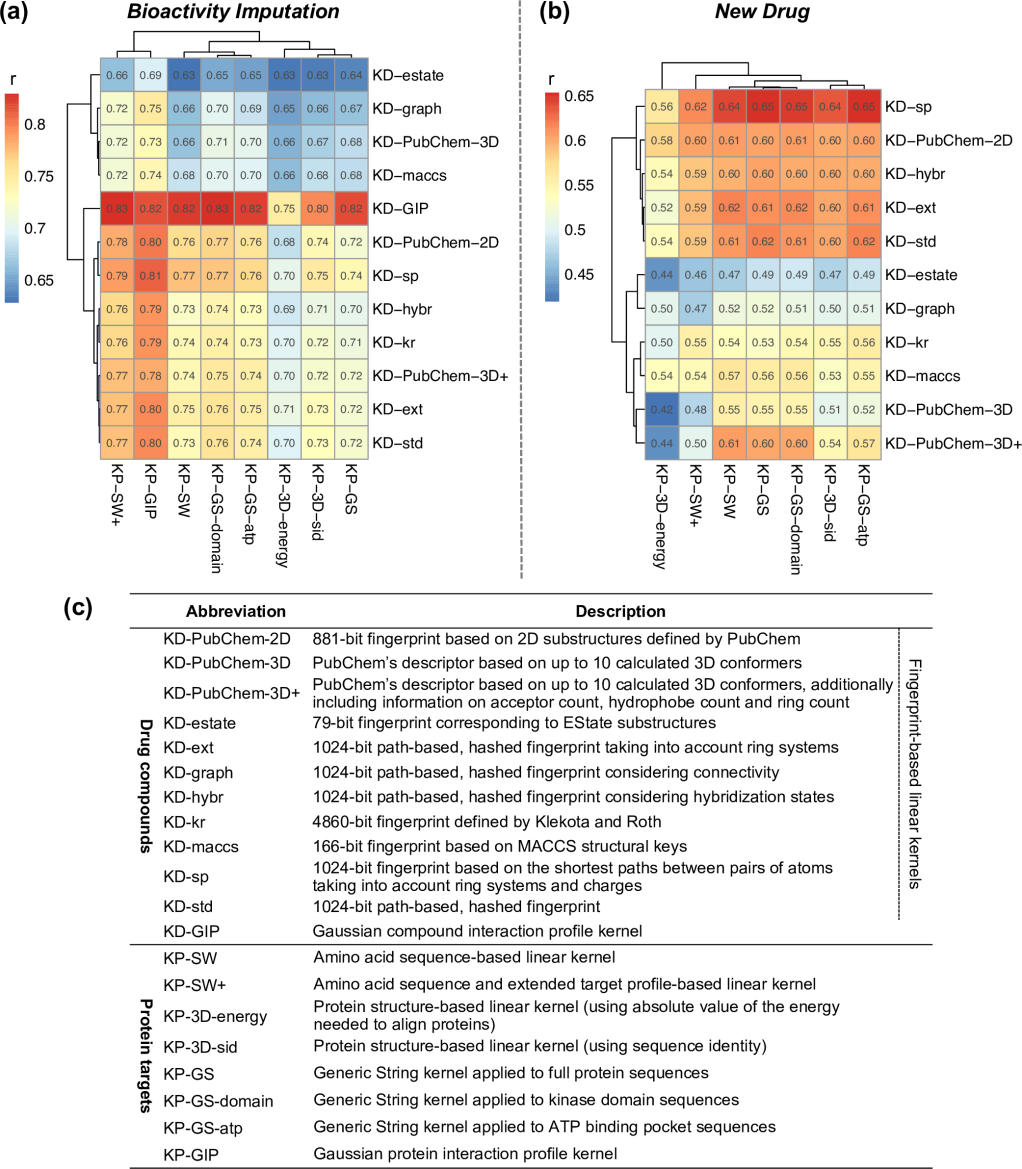
Computational evaluation of the model predictions. (**a**) Leave-one-out and (**b**) leave-drug-out cross-validation results. The prediction accuracy was evaluated with Pearson correlation (r) between binding affinities (pK_i_) from the study by Metz *et al*. [3] and those predicted using KronRLS algorithm with different pairs of compound (rows) and protein (columns) molecular descriptors encoded as kernel matrices (**c**). The corresponding root mean squared error (RMSE) values are shown in S1 Fig. Of note, Gaussian interaction profile drug kernel (KD-GIP), which resulted in the highest predictive performance under the *Bioactivity Imputation* scenario (a), was not evaluated under the *New Drug* scenario (b), because it is constructed based on the bioactivity profile of a drug to be predicted, that is, using information that in practice is unavailable when predicting target interactions for a new investigational drug compound.

In the first task of filling the gaps in experimental bioactivity profiling study, the Gaussian interaction profile drug kernel (KD-GIP) clearly outperformed other compound descriptors (Fig 3A and S1 Fig). This is because, in some cases, even a minor structural difference between chemical molecules causes a striking change in their potency, and such kernel is able to capture this behavior. Among structural fingerprint-based drug kernels, the ones constructed by comparison of two-dimensional substructures defined by PubChem (KD-PubChem-2D) as well as shortest paths between atoms (KD-sp) yielded the best binding affinity predictions. In case of both compounds and proteins, the use of three-dimensional conformations (KD-PubChem-3D, KP-3D-sid, KP-3D-energy) did not lead to improved prediction results.

Among the protein kernels, the protein interaction profile kernel (KP-GIP) and the kernel based on extended target profile built upon Smith-Waterman amino acid sequence comparisons (KP-SW+) showed the best overall performance (Fig 3A). Moreover, KP-SW+, paired with any drug kernel, achieved higher predictive accuracy than its commonly used counterpart KP-SW, which is also based on the Smith-Waterman amino acid sequence alignments but of only proteins included in the training data set, whereas KP-SW+ kernel is calculated based on more comprehensive, global features (see Materials and Methods for details). Notably, generic string kernel worked better with kinase domains (KP-GS-domain) and ATP-binding pockets (KP-GS-atp), compared to full amino acid sequences (KP-GS), indicating their potential for compound-target interaction inference.

Taken together these computational evaluation results under the *Bioactivity Imputation* scenario, the best chemical and genomic molecular descriptor pair in filling the gaps in experimental kinase inhibitor target profiling study was formed by KD-GIP and KP-GS-domain kernels, followed closely by KD-GIP and KP-SW+ kernels, which resulted in high Pearson correlations between the original and predicted compound-kinase binding affinities of 0.829 and 0.828, respectively (p < 0.0001, S2 Fig).

#### *New Drug*: Computational evaluation of predicting target interactions for new compounds

Predicting bioactivity signatures for a new drug candidate is a lot more challenging task, since such chemical probe has no target interaction data available for the model training. To simulate this setup, we next performed LDO-CV using different pairs of chemical and genomic molecular descriptors, but without including interaction profile kernels. Among drug kernels, the one computed using the shortest paths between atoms (KD-sp) demonstrated the best overall performance (Fig 3B). Together with amino acid sequence-based generic string protein kernel (KP-GS), it formed the most powerful molecular descriptor pair in the inference of interactions for an investigational drug candidate, achieving Pearson correlation of 0.653 (p < 0.0001, S2 Fig). We note that a very similar predictive performance was achieved by KD-sp drug kernel coupled either with KP-GS-atp or KP-GS-domain protein kernels (Pearson correlations of 0.651 and 0.649, respectively).

These computational evaluation results demonstrate that an optimal choice of both drug and protein kernels depends on the practical application use case, such as whether one is interested in the *Bioactivity Imputation* or *New Drug* prediction scenarios. Computational CV protocols provided us useful tools for optimizing the prediction models, which is a critical prerequisite for the achievement of high-quality binding affinity predictions, before going into the more laborious and expensive experimental validations.

### Filling the experimental gaps in large-scale kinase inhibitor target profiling study

Next, we trained the KronRLS algorithm with 16,265 bioactivities between 152 kinase inhibitors and 138 kinases measured in the study by Metz *et al*. [3], together with the best-performing under the *Bioactivity Imputation* scenario drug interaction profile kernel (KD-GIP) and kinase domain-based generic string protein kernel (KP-GS-domain). We then used the optimized model to predict the remaining 4,711 binding affinities that were missing in this experimentally-measured compound-kinase interaction map (S9 Fig).

To assess the model’s practical utility, we experimentally tested a set of 100 predicted binding affinities between 5 drug compounds (cediranib, lapatinib, gefitinib, pazopanib and vx-745) and 20 kinases (*ABL1*, *AXL*, *BRK*, *BTK*, *EGFR*, *FAK*, *FYN A*, *HER2*, *HER4*, *IGF1R*, *InsR*, *ITK*, *JAK3*, *KDR*, *LCK*, *LYN B*, *PYK2*, *SRC*, *SYK*, *TRKA*). Among these, new potential interactions, not present in the Metz *et al*. dataset, were predicted for cediranib, lapatinib and gefitinib (S4 Fig). We note that pazopanib presented very high prediction accuracy, despite of having a sparse binding affinity profile available for the model training. On the other hand, vx-745 had no potent activities, either measured or predicted, against any of the kinases, and therefore it served as a negative control in the validation (S4 Fig). We tested the predicted bioactivities using a cell-free ADP-Glo Kinase Assay (see Materials and Methods for details).

**Fig 4:**
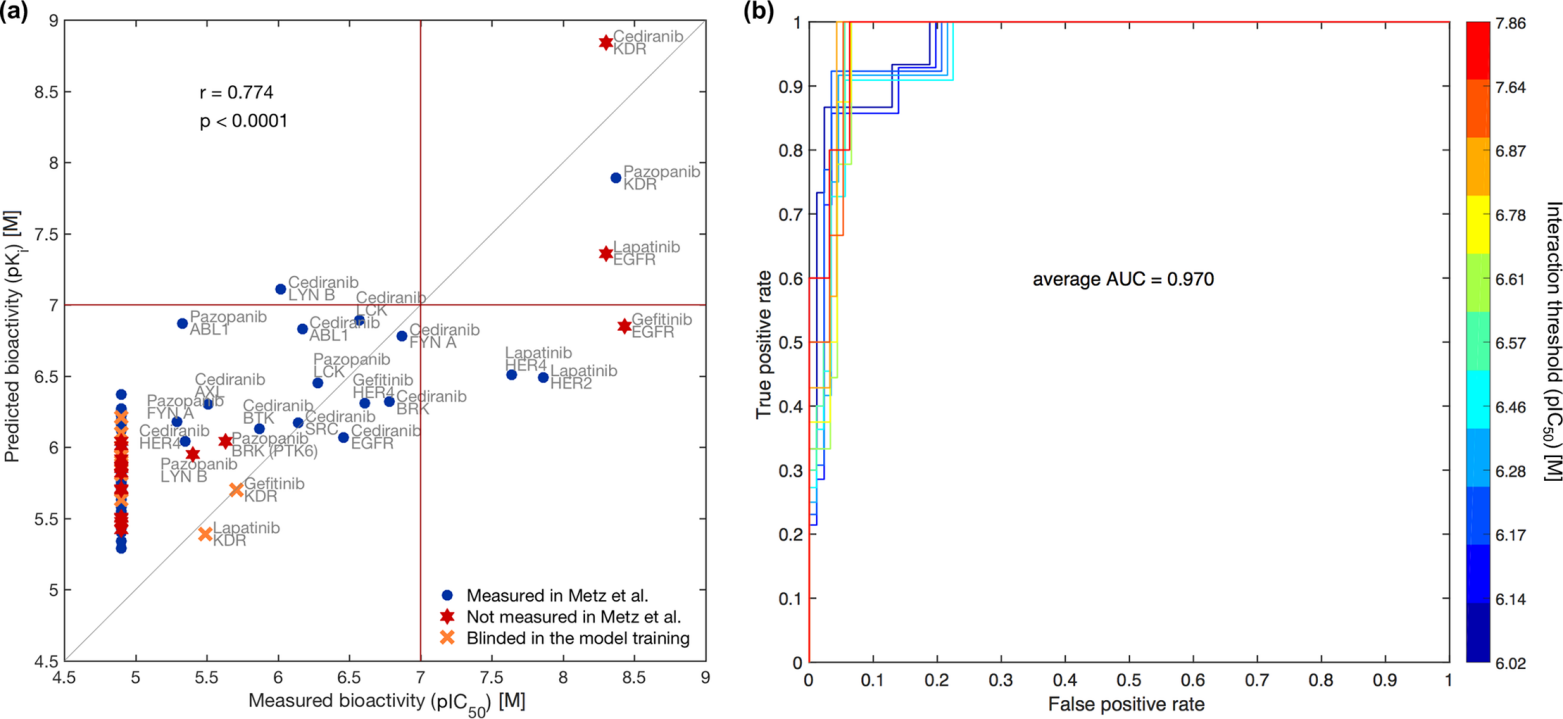
Comparison between computationally-predicted and experimentally-measured bioactivities. (**a**) Scatter plot between bioactivity values of 100 compound-kinase pairs (detailed in S2 Table). r indicates Pearson correlation. The orange cross points correspond to compound-kinase pairs tested in the study of Metz *et al*. but randomly blinded by us in the training of the model, forming an additional validation set. When no clear interaction between compound and kinase was observed in our experimental assay, the pIC_50_ value was set to 4.9 M, corresponding to the highest drug concentration used in our screen (12,500 nM). The higher the pK_i_/pIC_50_ value, the stronger the affinity between the two molecules. Red lines mark a relatively stringent interaction threshold (7 M), distinguishing the top left corner as the region containing false positive interaction predictions, and the bottom right corner as false negative predictions. (**b**) A set of receiver operating characteristic (ROC) curves to investigate the model performance as a function of varying activity threshold. We applied 11 different interaction threshold values from the pIC_50_ interval [6 M, 8 M] to binarize the experimentally-measured bioactivities into true class labels, and then determined how accurately the model can discriminate between the interacting and non-interacting compound-kinase pairs. The average area under the ROC curves (AUC) equals 0.970.

We observed a relatively high Pearson correlation of 0.774 (p < 0.0001) between the model-predicted (pK_i_) and experimentally-measured (pIC_50_) bioactivities among the 100 compound-kinase pairs (Fig 4A). The IC_50_ readout from our assay, similarly to the inhibition constant K_i_ in the Metz *et al*. study, indicates the concentration of the compound needed to inhibit enzymatic activity of a kinase by 50%. Even though IC_50_ is known to depend on the concentration of the enzyme, inhibitor, and substrate, along with other experimental conditions, whereas K_i_ is an intrinsic, thermodynamic quantity independent of the substrate [36], recent studies have shown a sufficiently high level of association between pIC_50_ and pK_i_ readouts, permitting their reliable comparison [37,38]. We also observed a strong technical correlation of 0.769 (p < 0.0001) between pIC_50_ readouts from our profiling assay and pK_i_ values measured in the study by Metz *et al*. (S5 Fig), which supports the feasibility of our experimental validations. Furthermore, based on the published information [39], the ATP concentration used in our assay (10 μM) is expected to be below, or in some cases equal to, the ATP K_m_ values of the kinases tested, suggesting that the IC_50_ values should be very close to the respective K_i_ values.

We also compared both the model-predicted and experimentally-measured interaction mapping to the results from another large-scale binding assay by Davis *et al* [40]. In this study, 72 clinically relevant kinase inhibitors were profiled against 442 kinases, providing, for each compound-kinase pair tested, dissociation constant K_d_, indicating the tendency of a larger molecular complex to dissociate reversibly into the component molecules. We again observed a very good agreement (correlation of 0.796, p < 0.0001) between the computationally-predicted (pK_i_) and measured (pK_d_) binding affinities across the overlapping 95 compound-kinase pairs (S5 Fig). We noted even a higher technical correlation of 0.916 (p < 0.0001) between pK_d_ values from Davis *et al*. study and pIC_50_ values from our experimental assay (S5 Fig). Notably, a comparison between predicted pK_i_ values and measured pK_d_ readouts across a larger set of 2,662 compound-kinase pairs overlapping between Metz *et al*. [3] and Davis *et al*. [40] studies resulted in a lower correlation (0.642, p < 0.0001, S6 Fig), compared to that when considering only the pairs included in our experimental assay (0.796, p < 0.0001, S5 Fig). A high pK_d_ indicates that a substrate is more likely to be bound to an enzyme, whereas pK_i_ measures the potency of a drug. Even though both high pK_i_ and pK_d_ values are considered as indicators of drug activity, a drug with high pK_i_ does not necessarily result in a high pK_d_ (S6 Fig).

As expected, the correlation between the model-predicted and measured by Metz *et al*. pK_i_ values in the training data (excluding pairs blinded in the model training, marked with an orange cross points in Fig 4A) was somewhat higher than that for the missing compound-kinase pairs (correlation of 0.802, p < 0.0001, S5 Fig). Of note, our experimental assay confirmed computationally-predicted high binding affinities between cediranib-*KDR*, lapatinib-*EGFR* and pazopanib-*KDR*, the two first of which were even not measured in the study of Metz *et al*. Further, although some bioactivities missing in Metz *et al*. dataset corresponded to compound-kinase pairs already tested in other studies (e.g. lapatinib-*EGFR* measured in the assay by Davis *et al*.), these were not used in the training of our model. Taken together, the observed high agreement between the predicted and experimentally-measured bioactivities demonstrates the potential of the kernel-based modeling framework with appropriately chosen kernels for filling the gaps in existing compound-target interaction maps.

### Prediction of off-target interactions for an investigational kinase inhibitor tivozanib

Finally, we tested whether the optimized model can also predict target interactions for a new chemical probe, which has no profiling data available for the model training. We used here tivozanib as an example of an investigational tyrosine kinase inhibitor, known to be potent towards all three VEGF receptors (*FLT1*, *KDR*, *FLT4*) [41]. Beyond VEGFRs, however, the target profile of tivozanib has otherwise remained poorly characterized, including its potential off-targets. We therefore again used 16,265 binding affinities between 152 compounds and 138 kinases measured in the study by Metz *et al*. to train the KronRLS model with shortest paths between atoms-based drug kernel (KD-sp) and amino acid sequence-based generic string protein kernel (KP-GS), which were found to perform best under the *New Drug* scenario. Since the model should always be tuned separately under distinct prediction scenarios, even if the training dataset is the same, the model used here and the one described in the previous section differ in their chosen kernels and optimized value of the regularization parameter. With the optimized model, we predicted the bioactivity of tivozanib against the set of 138 kinases (S3 Table).

**Fig 5:**
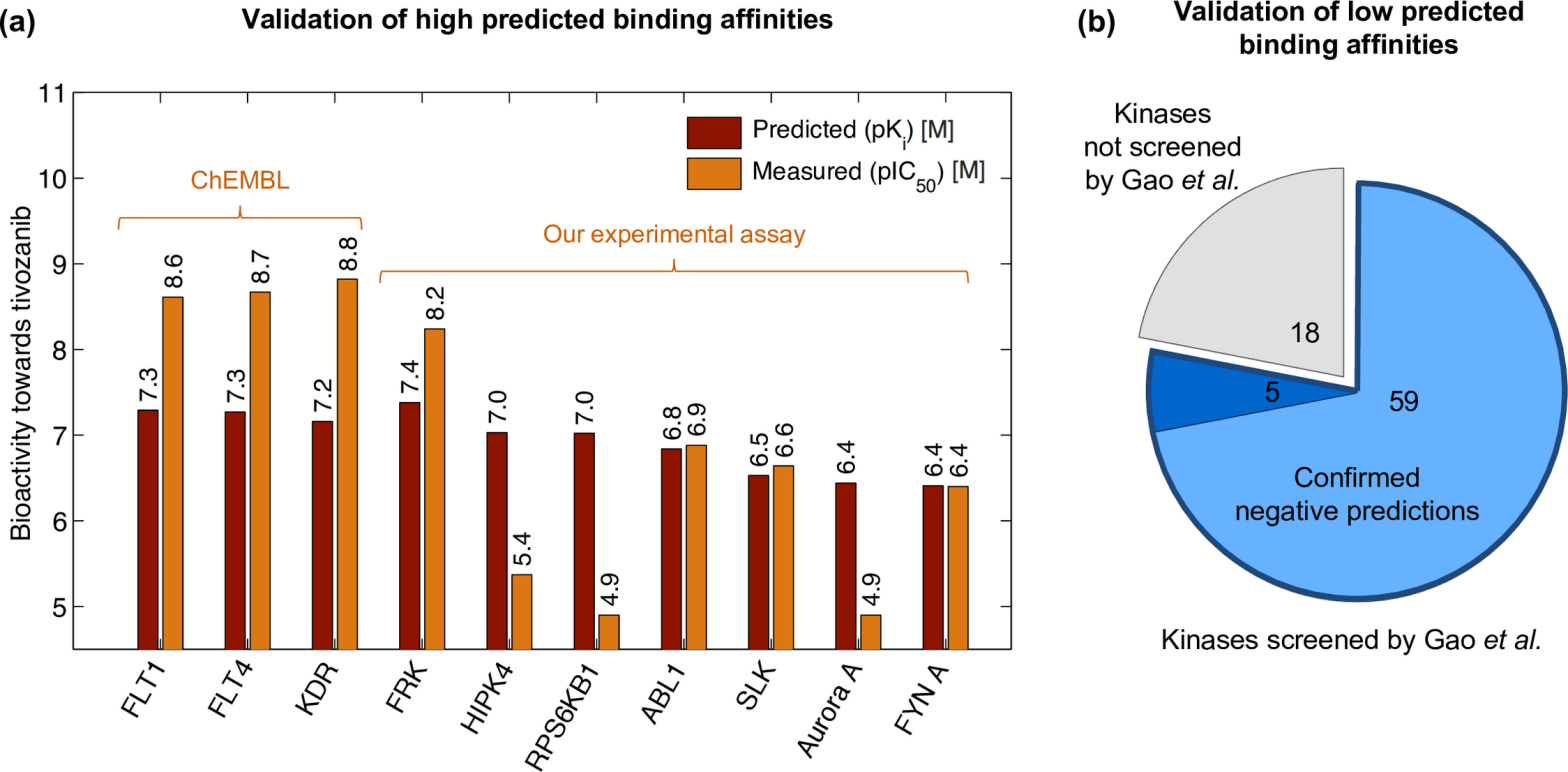
Prediction of target interactions for an investigational kinase inhibitor tivozanib. **(a)** Predicted and measured bioactivity profiles of tivozanib against its 3 established on-targets (*FLT1*, *FLT4*, *KDR*; average bioactivity from ChEMBL; S3 Table) and 7 predicted off-target kinases tested in our experimental assay. Pearson correlation r = 0.668 (p = 0.035). When no clear compound-kinase interaction was observed in our assay, the pIC_50_ value was set to 4.9 M, corresponding to the highest drug concentration used (12,500 nM). Predicted values belong to approximately constant range because we focused on experimental validation of the model-predicted off-target interactions. Three of them turned out to be false positives, and therefore the range of experimental results varies more than the range of predicted values. **(b)** Evaluation of negative interaction predictions from the model. Among 82 kinases with low predicted binding affinities (pK_i_ < 6 M), 64 were screened by Gao *et al*., and 59 of these are not likely targets of tivozanib (as they have at least 50% of the activity remaining at the high compound concentration of 1 μM).

As the first positive control, the model correctly predicted high potency of tivozanib against its known on-targets *FLT1*, *KDR* and *FLT4* (Fig 5A and S3 Table). To further assess the quality of the predictions, we used publicly available bioactivity data from the study of Gao *et al*. who profiled 158 kinase inhibitors, including tivozanib, for their inhibitory activity at 1 μM and 10 μM against 234 kinases [42]. Although the concentrations adopted in this screen were too high for pre-clinical testing of positive interactions, we used these data to evaluate the negative predictions from the model. In total, 64 out of 82 kinases with low predicted affinities (pK_i_ < 6 M) were screened by Gao *et al*. Among these, 59 kinases (92%) have at least 50% of the activity remaining at the high compound concentration of 1 μM (S3 Table), thus effectively validating the model’s negative predictions (Fig 5B).

We next went on and tested experimentally 7 predicted off-target interactions (*ABL1*, *Aurora A*, *FRK*, *FYN A*, *HIPK4*, *RPS6KB1*, *SLK*). These 7 kinases were selected among the set of 25 kinases with the highest predicted binding affinities by focusing on off-targets unique to tivozanib. Specifically, we compared the predicted target interaction profile of tivozanib to other VEGFR inhibitors found in the ChEMBL database [43]. For instance, *RET* was not selected, even though it was predicted to have high potency towards tivozanib (pIC_50_ = 6.9 M), since it is targeted by 76% of VEGFR inhibitors in ChEMBL (potency of at most 100 nM), whereas *FYN A* was included in our experimental assay because it is targeted only by 26% of VEGFR inhibitors (S3 Table). We tested the predicted bioactivities using a cell-free ADP-Glo Kinase Assay (see Materials and Methods for details).

Among the pre-selected off-target predictions, our experiments confirmed strong binding affinity between tivozanib and 4 out of the 7 tested kinases (57%), namely *FRK*, *ABL1*, *SLK* and *FYN A* (Fig 5A). Statistical significance of this success rate depends on the underlying distribution of the true target space of tivozanib, which is unknown. However, if one assumes that no more than 18 of 138 considered kinases are actual targets of tivozanib (13%), then the observed overlap is significant (p < 0.05, hypergeometric distribution). The observed correlation of 0.668 (p = 0.035, Fig 5A) between the predicted and measured binding affinities can be considered relatively high, given the rather limited spectrum of kinases tested and the fact that instead of selecting the top predicted off-targets only, we focused on the set of kinases that were unique to tivozanib among 25 kinases with the highest predicted binding affinities against it. Our experimental results provide not only novel insights into the MoA of tivozanib, but also demonstrate how the *in silico* framework offers a cost-effective tool for prioritizing the most promising target interactions of an investigational compound for further experimental evaluation.

## Discussion

Recently, a lot of effort has been placed on the development of systems-based machine learning models that could aid drug discovery process in terms of providing cost-effective compound-target bioactivity predictions. Their main differences lie in the way how the models construct and treat molecular descriptors, and utilize various learning techniques, including those based on random forest [16,19,21], kernel learning [15,22,23,32], recommender systems [26], matrix factorization [20,25,26], Boltzmann machines [27], deep neural networks [16,17], logistic regression [28], learning to rank [29], and ensemble learning [30,31]. Although such models have been shown to perform well in cross-validation setups, their practical benefits still remain largely unknown due to the lack of systematic verification, using targeted experimental assays, carried out sub-sequent to the prediction phase. In model-guided mapping applications, the validation experiments are performed based on the model predictions. Such experimental validation setup effectively avoids any possible information leakage between the training and validation data, since the validation data does not exist at the time of making the predictions. The computational-experimental approach, implemented in this study, therefore makes it impossible to overfit the model to the training data. Here, we used the approach to evaluate the predictive power of a well-established kernel-based learning technique [33]. We chose this model family since kernel regression approaches have proved good performance in recent computational studies, including prediction of drug-target interactions [32], peptide-protein binding affinities [44], drug sensitivities in cancer cell lines [45], as well as in metabolite identification [46] and QSAR modelling [35].

Taking into account that molecular interactions are not simple on/off relationships, we focused on a binding affinity prediction problem, using the RLS regression model with a Kronecker product kernel (KronRLS). Our systematic evaluation of the predictive performance of various descriptors in the form of kernels revealed that their choice has a critical impact on the prediction accuracy. This is expected because kernel matrix is a central component of the kernel-based learning algorithm as it should capture our prior belief on the relationships between the input objects. In particular, known binding affinities, even if sparse, constitute an important information source not only for model training but also for the kernel matrix construction. Purely structure-based chemical descriptors were not able to fully capture the changes in compounds’ activity caused by minor structural differences. Furthermore, we introduced a novel protein kernel (KP-SW+), based on extended target profile, and showed how it consistently outperformed its commonly-used counterpart, in which the Smith-Waterman amino acid sequence alignment is adapted exclusively to proteins included in the dataset of interest (KP-SW). This was evident particularly under the *New Target* setup (Fig 2C), where the aim is to predict compounds targeting a new protein not encountered in the training data (correlation of 0.669 for KP-SW+ vs. 0.506 for KP-SW; S7 Fig). Under the same setting, we also observed a clear advantage of using, for the first time in the context of drug-protein interaction inference, generic string kernel applied to kinase domains and ATP-binding pockets over full protein sequences (correlations of 0.651 for KP-GS-domain, 0.628 for KP-GS-atp, 0.508 for KP-GS; S7 Fig). The majority of kinase inhibitors, including those considered here, bind to ATP-binding pockets, and short sequences of these pockets are included within the kinase domain sequences, thus capturing also the neighbouring context. However, polypharmacological activities of kinase inhibitors, originating from the conservation of kinase ATP-binding pockets, make the prediction problem highly challenging, and better accuracies are likely obtained with compounds having more distinct target profiles. The methodology introduced here could equally well incorporate other compound and protein classes, such as ion channels or G-protein-coupled receptors (GPCRs), but further work will be required to investigate its practical performance under various scenarios using both computational and experimental validations. For instance, it remains an open question which kernels should be calculated to best represent such extended pharmacological spaces.

Our results also demonstrate the importance of a proper evaluation procedure of the *in silico* models. A rigorous computational CV protocol is critical to ensure realistic performance estimates for the optimized models. In particular, the lack of the nested CV strategy in the model selection may lead to over-optimistic prediction results [32]. It is also important that CV design reflects the practical application use case of the model. Given a query drug-protein pair (*d*_*x*_, *p*_*x*_), four different prediction scenarios can be distinguished, depending on whether there exist other compounds with measured bioactivities against *p*_*x*_, or proteins with measured bioactivities against *d*_*x*_ (Fig 2 and S1 Table). In turn, different types of CV designs need to be implemented in order to tune the model parameters and to evaluate its predictive performance. Here, we focused on the two most common and practical scenarios of the *Bioactivity Imputation* (Fig 2A) and *New Drug* (Fig 2B). Additionally, we provided the CV results under the *New Target* setup (Fig 2C) in S7 Fig. We first adopted LOO-CV, a design that simulates scattered missing values in otherwise known compound-target bioactivity map, to optimize the model and asses its performance in filling the experimental gaps (*Bioactivity Imputation*). Next, LDO-CV was applied in predicting target interactions for new candidate compounds having no measured bioactivity data for the model training (*New Drug*). The latter is much more challenging task, which was also demonstrated in our results; the correlation between the measured and predicted binding affinities under the *Bioactivity Imputation* setup was much higher (0.829) than under the *New Drug* scenario (0.653). However, even in the latter scenario, we still obtained a large number of statistically significant correlation values (S3 Fig). We further observed a high average AUC values under the *Bioactivity Imputation* setup (0.945, S2 Fig), but also under the *New Drug* prediction scenario (0.853, S2 Fig), which indicates that the model is able to discriminate well the interacting from non-interacting compound-kinase pairs. As expected, the classification accuracy increased with the increasing activity threshold as the true positive set includes a growing number of most likely interactions.

In practical applications, the method requires features extracted from both compounds and proteins, such as readily available chemical two-dimensional structures and amino acid sequences, respectively, based on which kernels can be then calculated. However, if one is interested in a bioactivity of an investigational drug against a protein with unknown sequence, a reasonable prediction accuracy can still be achieved if there exist other compounds with measured bioactivities against a query protein (the *New Drug* setup). The practical solution is to replace the protein kernel matrix with an identity matrix, which implies that each protein will be considered similar to itself only and, effectively, the model will use just known bioactivity data and drug-drug similarities during both training and prediction phase. In particular, we noted only a small drop in Pearson correlation after replacing the protein kernel (KP-GS) with the identity matrix (0.645 vs. 0.653). This observation is expected in multitask or transfer learning problems, such as the *New Drug* or *New Target* setups, where one of the similarities is essential for generalizing to new instances (drug-drug similarities under the *New Drug* setting, protein-protein similarities under the *New Target* scenario).

Ultimately, even though proper CV design is crucial to tune the model and assess its performance, subsequent experimental verification in the laboratory is the only way to really demonstrate the practical utility of the model predictions for drug discovery applications. The relatively good agreement between the computationally-predicted and experimentally-measured bioactivities validated the potential of the kernel-based algorithm, not only for filling the experimental gaps in existing drug-target interaction maps, but also in later stages of the drug development process, including prioritizing new target interactions of investigational compounds for further experimental evaluation, hence assisting in understanding of their MoA. Even though *in silico* inference of target interactions for new candidate drug compounds is a highly challenging task, our results with tivozanib suggest that, given enough-representative and high-quality training data, reliable off-target interaction predictions can be made. In addition to tivozanib, we initially considered also three other investigational kinase inhibitors, namely fedratinib, vx11e and ulixertinib (a compound derived from vx11e). However, we finally selected tivozanib because, unlike for the other compounds, its known on-targets were placed among the strongest predicted target interactions (S3 Table). This indicates that the primary on-target space of tivozanib is well-represented in our training data (S8 Fig). In the future, it is therefore important to profile and build up more diverse training data sets, including more examples of compounds targeting different kinase and other target classes.

Tivozanib was originally-developed as a VEGFR inhibitor meant to block angiogenesis by targeting endothelial cells in the tumor vasculature. However, its MoA has not yet been fully elucidated. Based on the model predictions, our experimental assay confirmed the two Src family kinases *FRK* and *FYN A*, as well as the non-receptor tyrosine kinase *ABL1* and serine/threonine kinase *SLK* as tivozanib’s off-targets. Our results highlight that tivozanib has an unusual target spectrum beyond the VEGFR family of kinases, and this suggests that the best anti-cancer use of this compound may not be in diseases where other VEGFR inhibitors with different target profiles have proven effective, but rather in ones where the target spectrum of tivozanib is more unique. For example, it can be hypothesized that tivozanib may have powerful activity in Src-family kinase addicted cancers, where it would target both angiogenesis and the cancer cells directly. Tivozanib has been shown to have a better safety profile than other marketed tyrosine kinase inhibitors and, currently, it is undergoing several clinical trials for the treatment of renal cell carcinoma (NCT03136627), refractory advanced renal cell carcinoma (NCT02627963), metastatic and non-resectable soft tissue sarcomas (NCT01782313), advanced liver cancer (NCT01835223), recurrent ovarian, fallopian tube, or primary peritoneal cancer (NCT01853644), and advanced prostate cancer (NCT01885949; July 2017). It will be interesting to see which of these trials will report successful treatment outcomes, and which will be terminated due to insufficient efficacy or toxicity.

Although presented here results are promising, there is much room for improvement. For instance, we formulated the predictive model using only one pair of chemical and genomic descriptors at a time. However, even better accuracies could be obtained with a multiple kernel learning framework, which integrates multiple biological and molecular data sources, along with learning their importance for the prediction task [47]. Additional improvement of the predictive performance could be achieved also by creating more sophisticated kernelized molecular descriptors, for instance, by comparing three-dimensional structures of protein binding pockets. Furthermore, we used here as the training data a single yet very comprehensive kinase inhibitor profiling assay containing large number (16,265) of measured compound-kinase binding affinities, spanning different kinase branches (S11 Fig). However, as a future direction, we plan to work on integrating bioactivity values originating from various target profiling experiments and bioactivity end-points into a single model [3,19,40,48]. Recently-initiated community-driven efforts, such as Drug Target Commons (https://drugtargetcommons.fimm.fi), which aim to collectively extract, manage and curate high-quality compound-target bioactivity data from public databases, literature and other resources, as well as annotate them with a common ontology, will be essential to facilitate the data standardization and computational modelling purposes. Nevertheless, we hope the current work provides a useful starting point and a practical guide on how to computationally prioritize the most promising target interactions for further experimental evaluation.

## Materials and methods

### Bioactivity dataset

We used publicly available compound-target interaction map generated by Metz *et al*.using a large-scale functional bioassay, which measured the concentration of a compound needed to inhibit the reaction catalysed by a kinase enzyme of interest by 50% [3]. The readout corresponds to an inhibition constant K_i_, typically expressed in the logarithmic scale as pK_i_ = -log_10_K_i_. Although the universal activity threshold cannot be explicitly defined for each compound-kinase pair, the higher the pK_i_ value, the stronger the binding affinity between the compound and kinase.

Among molecules included in the screen, 201 compounds are present in ChEMBL [43], and 169 proteins belong to the group of catalytically active human protein kinases [37]. The study is not complete, and therefore we used here a subset of these data: kinases and compounds for which at least 30% of the binding affinity values were measured, resulting in 152 drug compounds and 138 kinase targets. In total, there are 16,265 binding affinities in this selected interaction map (S9 and S10 Figs). Of note, most of the compounds constitute investigational, not yet FDA-approved, chemical probes.

### Prediction model

In supervised learning tasks, training data has the form ${\left\{(x_{i},y_{i})\right\}}_{i=1}^{N}$, where *N* denotes the number of training examples, **x**_*i*_ ∈ *X* is an input object represented as a vector with the feature values (e.g. a compound represented as a fingerprint vector) and *y*_*i*_ ∈ *Y* is its known associated label value (e.g. a potency of a compound against a certain protein). The aim is to find a prediction function *f* that models the relationship between **x**_*i*_’s and *y*_*i*_’s, and which can then be used to predict the label values for new instances outside the training space. Classical algorithms search for linear dependencies but often the actual relations underlying the data are highly nonlinear. Kernels offer the advantage of increasing the power of the linear learning machines by providing a computationally efficient way of projecting the input data into a high-dimensional feature space. A linear model in this implicit feature space corresponds to a nonlinear model in the original space. A separation of the statistical learning technique and the data representation is another convenient attribute of kernels.

Formally, a kernel is a function *k* that for all **x**,**z** ∈ *X* satisfies *k*(**x**,**z**) = ⟨***ϕ***(**x**),***ϕ***(**z**)⟩, where *ϕ* is a mapping from the input space *X* to an inner product high-dimensional feature space *F*: ***ϕ***: **x** ∈ *X* → ***ϕ***(**x**) ∈ *F*, and it can be considered as a similarity measure between two objects **x** and **z**. It is, however, often possible to avoid the explicit computation of the mapping *ϕ*, and define the kernel directly in terms of the original input data items by replacing the inner product ⟨∙,∙⟩ with an appropriately chosen kernel function satisfying certain mathematical properties (so-called kernel trick). Kernels are particularly handy for calculating similarities between structured objects, including molecules.

Here, we focused on a regression problem with the objective of predicting real-valued compound-target binding affinities. We used Kronecker regularized least-squares model (KronRLS) [33,49], a special variant of kernel ridge regression (KRR) which combines linear least squares with L2-norm regularization (ridge regression) and the kernel trick [50].

In KRR, given a set of *N* compound-protein pairs as training inputs **x**_*i*_’s, *i* = 1,…, *N*, and associated labels *y*_*i*_’s indicating binding affinities between them, we aim to find the minimizer of the following objective function *J*:

$$J(f)=\sum_{i=1}^{N}({f(x_{i})-y}_{i}{)}^{2}+\lambda {\left\|f\right\|}_{k}^{2},$$ (1)

where *f* indicates the prediction function, *f*(**x**_*i*_) is the predicted binding affinity of *i*^th^ compound-protein pair **x**_*i*_, *λ* denotes a regularisation parameter controlling the balance between training error and model complexity (*λ* > 0), and ‖*f*‖_*k*_ is a norm of *f* on the space associated to kernel function *k*. In Eq (1), the first term corresponds to the training error, and the second, controlled by *λ*, is the penalty term that is larger for complex models that are more likely to overfit to training data but not generalize well to new instances.

According to the representer theorem [51], the prediction function that minimizes *J(f)* can be expressed in terms of linear combination of the training examples:

$$f(x)=\sum_{i=1}^{N}\alpha_{i}\;k(x_{i},x)=\alpha^{T}k,$$ (2)

where **k** is a vector with kernel values *k*(**x**_i_,**x**) between each training point **x**_*i*_ and test point **x** for which the prediction is made. The squared norm of *f* is therefore written as

$${\left\|f\right\|}_{k}^{2}=\sum_{i=1}^{N}\sum_{j=1}^{N}\alpha_{i}\;\alpha_{j}\;k(x_{i},x_{j}).$$ (3)

A vector **α**, consisting of parameters *α*_*i*_ that define the solution to KRR, is found by solving the following system of linear equations

(K+λI)α=y, (4)

where **I** is the *N*×*N* identity matrix, and **y** is the vector consisting of labels *y*_*i*_. **K** denotes *N*×*N* pairwise kernel matrix constructed for all training examples **x**_*1*_, **x**_*2*_,…, **x**_*N*_, and thus containing similarities between all compound-protein pairs. However, the size of **K** makes the training of the model computationally very heavy, even for moderate number of compounds and proteins.

KronRLS is a special variant of KRR, where one assumes each data point **x**_*i*_to consist of two separate parts, such as compound and protein, each equipped with its own kernel function, which enables to speed up the model training. Indeed, pairwise kernel **K** is computed as the Kronecker product of compound kernel **K**_*D*_ of size *n*_*D*_×*n*_*D*_ and protein kernel **K**_*P*_ of size *n*_*P*_×*n*_*P*_ (*N* = *n*_*D*_×*n*_*P*_):

$$K=K_{D}⨂K_{P}.$$ (5)

Using Eq (5), the solution to KronRLS can be calculated from:

$$\alpha =\mathrm{vec}(U_{P}CU_{D}^{T}),$$ (6)

where vec(·) is the vectorization operator that arranges the columns of a matrix into a vector, **U**_*D*_ and **U**_*P*_ are orthogonal matrices with eigenvectors of drug kernel **K**_*D*_ and protein kernel **K**_*P*_, respectively:

$$K_{D}=U_{D}\Sigma_{D}U_{D}^{T},$$ (7)

$$K_{P}=U_{P}\Sigma_{P}U_{P}^{T},$$ (8)

and **C** is a matrix for which it holds that:

$$\mathrm{vec}(C)={(\Sigma }_{D}⨂\Sigma_{P}+\lambda I{)}^{-1}\mathrm{vec}(U_{P}^{T}Y^{T}U_{D}).$$ (9)

Here, **Σ**_*D*_ and **Σ**_*P*_ denote diagonal matrices containing eigenvalues of **K**_*D*_ and **K**_*P*_. Label matrix **Y** stores binding affinities between *n*_*D*_ drug compounds (rows) and *n*_*P*_ protein targets (columns). This way, we completely avoid the computation of the large pairwise kernel **K**, and therefore significantly shorten the training time. After applying the well-known property of the Kronecker product, (**A⊗B**)vec(**D**) = vec(**BDA**^*T*^), the prediction for test point **x** can be calculated as

$$f(x)=(k_{D}⨂k_{P})\alpha =(k_{D}⨂k_{P})vec(U_{P}CU_{D}^{T})=k_{P}(U_{P}CU_{D}^{T})k_{D}^{T}.$$ (10)

Of note, the above shortcuts work only if there are no missing values present in the label matrix **Y**. Thus, as the pre-processing step, we imputed the missing binding affinities in **Y** by the weighted row (compound) average. The contribution of each protein was weighted by its similarity (normalized Smith Waterman score) to the protein for which the binding affinity was missing. Such imputed values were discarded when assessing the predictive performance of the model.

The implementation of KronRLS is available at https://github.com/aatapa/RLScore. We tuned the regularization parameter *λ* of KronRLS algorithm using nested CV (Table 1).

### Molecular descriptors

We computed several types of drug compound and protein target molecular descriptors in the form of kernel matrices **K**_*D*_ and **K**_*P*_, respectively. The summary is presented in Fig 3C.

#### Drug compound space

For drug compounds, we calculated 11 fingerprint-based linear kernels and Gaussian interaction profile kernel.

Fingerprint encodes a chemical structure into a binary vector, where each bit represents the presence (1) or absence (0) of the specific substructure in the molecule. We compared two drug compounds **d**_*i*_ and **d**_*j*_, represented by their fingerprints, using Tanimoto similarity score, calculated based on the size of common substructures of the molecules:

$$S_{D}(d_{i},d_{j})=\frac{N_{{fp}_{i}{,fp}_{j}}}{N_{{fp}_{i}}+N_{{fp}_{j}}-N_{{fp}_{i}{,fp}_{j}}},$$ (11)

where *fp* denotes the fingerprint, *N*_*fpi*_ is the number of 1-bits in the fingerprint *fp*_*i*_ of compound **d**_*i*_, and *N*_*fpi*,*fpj*_ indicates the number of 1-bits in fingerprints of both compounds **d**_*i*_ and **d**_*j*_. The idea behind Tanimoto score is based on Jaccard’s index, commonly used for comparing sample sets. Given a matrix **S**_*D*_ of Tanimoto scores between compounds, we computed the fingerprint-based linear drug kernel as:

$$K_{D}=S_{D}S_{D}^{T}.$$ (12)

We used the following 11 types of fingerprints calculated using Chemical Structure Clustering Tool of PubChem [52] (http://pubchem.ncbi.nlm.nih.gov) and rcdk package available for R [53].

- **D-PubChem-2D**: 881-bit fingerprint based on two-dimensional substructures defined by PubChem.
- **D-PubChem-3D**: PubChem’s descriptor based on up to 10 calculated three-dimensional conformers of the compound.
- **D-PubChem-3D+**: PubChem’s descriptor based on up to 10 calculated three-dimensional conformers of the compound, including additional information (acceptor count, hydrophobe count, ring count etc.).
- **D-std**: 1024-bit path-based, hashed fingerprint.
- **D-ext**: 1024-bit path-based, hashed fingerprint taking into account ring systems.
- **D-graph**: 1024-bit path-based, hashed fingerprint considering connectivity.
- **D-hybr**: 1024-bit path-based, hashed fingerprint considering hybridization states.
- **D-sp**: 1024-bit fingerprint based on the shortest paths between pairs of atoms taking into account ring systems and charges.
- **D-estate**: 79-bit fingerprint corresponding to Estate substructures described by Hall and Kier [54].
- **D-maccs**: 166-bit fingerprint based on MACCS structural keys developed by MDL Information Systems [55].
- **D-kr**: 4860-bit fingerprint defined by Klekota and Roth [56].

Furthermore, we defined a feature vector for each drug compound as its quantitative interaction profile, i.e. a feature vector **v**_*i*_ contains binding affinities (pK_i_ values) between compound **d**_*i*_ and *n*_*P*_ proteins present in our data set, i.e. **v**_*i*_ = **Y**_*i·*_ (*i*^th^ row of **Y**). Due to data sparsity, we first imputed the missing bioactivity values in **Y** by the weighted row average, where each row corresponds to binding affinities of a single compound. The contribution of each protein was weighted by its similarity (normalized Smith Waterman score) to the protein for which the interaction value was missing. Then, we constructed a Gaussian kernel as follows:

$$K_{D-GIP}(d_{i},d_{j})=exp\left(-\frac{{\left\|v_{i}-v_{j}\right\|}^{2}}{2\sigma^{2}}\right),$$ (13)

where *σ* is the kernel width.

#### Protein target space

For kinase targets, we calculated 8 molecular descriptors, i.e. 4 linear kernels based on comparing protein amino acid sequences and three-dimensional structures, 3 generic string kernels applied to full amino acid sequences, kinase domains and ATP-binding pockets, as well as Gaussian interaction profile kernel.

We computed linear protein kernels as:

$$K_{P}=S_{P}S_{P}^{T},$$ (14)

where **S**_*P*_ is one of the four protein-protein similarity matrices (**S**_*P-SW*_, **S**_*P-SW+*_, **S**_*P-3D-energy*_, **S**_*P-3D-sid*_), described in the following.

In the first case, we compared protein amino acid sequences using normalized version of Smith-Waterman (SW) alignment score [57]:

$$S_{P-SW}(p_{i},p_{j})=\frac{SW(p_{i},p_{j})}{\sqrt{SW(p_{i},p_{i})}\sqrt{SW(p_{j},p_{j})}},$$ (15)

where **p**_*i*_ denotes *i*^*th*^ protein target, and *SW*(·,·) is the original Smith-Waterman score.

Moreover, we introduced a novel molecular descriptor based on *extended target profile*. In this case, we derived features for each protein target **p**_*i*_ present in our dataset by calculating normalized SW scores between **p**_*i*_ and the bigger set of 20,239 human proteins (**h**_*l*_’s) from the UniProt database [58] (http://www.uniprot.org/):

$$S_{P-SW+}(p_{i},h_{l})=\frac{SW(p_{i},h_{l})}{\sqrt{SW(p_{i},p_{i})}\sqrt{SW(h_{l},h_{l})}}.$$ (16)

Thus, given 138 protein targets in our data set, constructed **S**_*P-SW+*_ matrix has the size of 138 × 20,239. This procedure could be thought of in terms of multiple sequence alignment, and it allows to derive global protein target features. We used BLOSUM 50 matrix in all the experiments employing SW alignments.

In order to measure pairwise similarities between three-dimensional protein structures, we used MISTRAL software, which aligns two proteins based on the minimisation of an energy function over the low-dimensional space of the relative orientations of the molecules [59]. We downloaded kinase structures from the Protein Data Bank database [60] (PDB; http://www.rcsb.org/). PDB files were available for 109 out of 138 kinases in our data set; we retrieved the structures of the remaining 29 molecules based on their amino acid sequence homology to proteins present in the PDB database. Then, we calculated **S**_*P-3D-energy*_ and **S**_*P-3D-sid*_ similarity matrices relating all pairs of proteins, analogously as in Eq (15) but using obtained from MISTRAL absolute value of the energy needed to align proteins or the sequence identity, respectively, instead of the *SW*(·,·) scores. The sequence identity value refers to the number of aligned amino acids that are of the same chemical type.

We also compared protein targets using generic string (GS) kernel incorporating amino acid properties [44]. GS kernel compares each substring of protein sequence **s** of size *l* ≤ *L* with each substring of protein sequence **s’** having the same length:

$$K_{P-GS}(s,s\prime )=\sum_{l=1}^{L}\sum_{i=0}^{|s|-l}\sum_{j=0}^{|s\prime |-l}\exp\left(\frac{-{\left(i-j\right)}^{2}}{2\sigma_{p}^{2}}\right)exp(\frac{-{\left\|\psi^{l}(s_{i+1},\ldots ,s_{i+l})-\psi^{l}({s\prime }_{j+1},\ldots ,{s\prime }_{j+l})\right\|}^{2}}{2\sigma_{c}^{2}}).$$ (17)

Here, each comparison results in a score that depends on the shifting contribution term (difference in the position of two substrings in **s** and **s’**, controlled by *σ*_*p*_ parameter) and the similarity of amino acids included in two substrings (controlled by *σ*_*c*_ parameter). The kernel outputs the sum of the scores from all the substring comparisons.

Vector **ψ**^***l***^ is constructed as follows. Each type of amino acid *a*_*m*_, *m* = 1,…, *M*, (e.g. Asparagine) has a corresponding feature vector **ψ**(*a*_*m*_) which defines its *d* properties:

$$\psi (a_{m})=(\psi_{1}(a_{m}),\psi_{2}(a_{m}),\ldots ,\psi_{d}(a_{m})).$$ (18)

Given a protein sequence **s** = *s*_*1*_, *s*_*2*_,…, *s*_*l*_, with all *s*_*l*_ ∈ Σ, where Σ is the set of all *M* amino acids, **ψ**^***l***^(**s**) is its encoding function which concatenates *l* vectors describing each amino acid the sequence **s** is composed of:

$$\psi^{l}(s)=(\psi (s_{1}),\psi (s_{2}),\ldots ,\psi (s_{l})).$$ (19)

Furthermore, the shifting contribution term gives the GS kernel a useful property of enabling to match two amino acid subsequences even if their positions in the full protein sequences differ notably.

We used BLOSUM 50 matrix as amino acid descriptors, and we calculated three GS kernels based on comparing full protein kinase sequences, as well as sequences of kinase domains and ATP-binding pockets retrieved from the PROSITE database [61] (http://prosite.expasy.org/).

Finally, we calculated Gaussian protein interaction profile kernel as:

$$K_{P-GIP}(p_{i},p_{j})=exp\left(-\frac{{\left\|w_{i}-w_{j}\right\|}^{2}}{2\sigma^{2}}\right).$$ (20)

The formulation above is analogous to the compound interaction profile kernel defined in Eq (13), but now feature vector denoted by **w**_*i*_ contains binding affinities between protein **p**_*i*_ and all compounds present in our data set. All the kernel matrices are positive semidefinite and were normalized. Kernel parameters were tuned using nested cross-validation.

### Model evaluation procedure

We used nested cross-validation (CV) procedure for model selection, and we assessed the predictive power with Pearson correlation and root mean squared error (RMSE) between original and predicted pK_i_ values.

In *k*-fold CV, the dataset is randomly divided into *k* subsamples of equal size, and the model is trained based on *k*-1 of them (training data). Then, the remaining subsample (test data) is used to assess how well the model that has been found generalizes to new instances, i.e. to calculate the predictive performance. The procedure is repeated *k* times, such that each subsample is used once as the test data, and the average error over the *k* folds gives the final estimate.

Nested CV consists of two loops, outer and inner one. In the outer CV loop, each of the *k* folds is kept as a test set at a time. The remaining *k*-1 training folds of the outer CV loop are further divided into training and test set of the inner CV loop. Here, the inner CV was performed during each round of the outer CV, with the aim of selecting the regularization parameter *λ* of KronRLS algorithm as well as kernel parameters (S4 Table). We performed a grid search in order to select the most suitable combination of all parameters. Then, training folds of the outer CV loop were used to train the model with selected parameters, and the predictive performance was evaluated on the test set.

We applied two different CV strategies, i.e. leave-one-out nested cross-validation (LOO-CV) and leave-drug-out nested cross-validation (LDO-CV), summarized in Table 1 and S12 and S13 Figs.

We note that it is critical to discard binding affinities of compound-protein pairs belonging to the test fold of both inner and outer CV prior to computing Gaussian interaction profile kernels (KD-GIP, KP-GIP) in order to avoid significant model overfitting. Here, we used interaction profile kernels with LOO-CV, and we removed from the compound-protein interaction matrix **Y** the whole column (row) containing the test point before computing KD-GIP (KP-GIP) kernel.

### Kinase assays

#### Testing bioactivities predicted to fill the experimental gaps

We used Kinase Selectivity Profiling System + ADP-Glo Assay kits (Promega, TK-1 V6851, TK-2 V6853, TK-3 V6921) for performing kinase selectivity profiling at 1uL reaction volume on 1536-well assay plates (Corning, #3937). The reagents were prepared as indicated in Promega technical manual (TM421), and transferred to Echo Qualified 384-Well Polypropylene Microplate source plates (Labcyte, P-05525). We pre-plated five kinase inhibitors tested (cediranib, lapatinib, gefitinib, vx-745, pazopanib; all dissolved in DMSO; S5 Table) at 12 concentrations (12500, 3750, 1250, 375, 125, 37.5, 12.5, 3.75, 1.25, 0.375, 0.125, 0.0375 nM) on the 1536-well white round bottom assay plates using acoustic dispenser (Labcyte, ECHO550 Liquid handler) and DMSO transfer calibration. The inhibitors were transferred at 7.5 and 2.5 nL volumes from Echo Qualified 384-Well Low Dead Volume Microplates (Labcyte, LP-0200).

The kinase inhibitors were dissolved to 1X Kinase Buffer with 5% DMSO by transferring 200nL of the buffer using ECHO525 Liquid handler (Labcyte). After the transfer, the assay plate was immediately sealed to minimize evaporation, and centrifuged at 872rcf/2min/RT. The dissolved kinases and 2.5X kinase buffer controls were transferred at 400nL using ECHO525. Plates were sealed, centrifuged at 872rcf/2min/RT, mixed with plate shaker for 2 minutes and incubated 10min/RT plate upside down. The 2.5X ATP/substrate working stocks were transferred at 400nL using ECHO525. Plates were sealed, centrifuged at 872rcf/2min/RT, mixed with plate shaker for 2 minutes, and incubated upside down (60min/RT).

For the ADP detection, 1 μl ADP-Glo reagent was dispensed (Thermo Scientific Multidrop Combi nL). Plates were sealed, centrifuged at 872rcf/2min/RT, mixed with plate shaker for 2 minutes and incubated upside down (40 min/RT). Next, 2 μl kinase detection reagent was dispensed with Thermo Scientific Multidrop Combi nL. Plates were sealed, centrifuged at 872rcf/2min/RT, mixed with plate shaker for 2 minutes and incubated upside down (30min/RT). Luminescence (0.5sec/well) was measured using PheraSTAR (BMG LABTECH) and 1536-plate compatible spoon.

The Kinase Selectivity Profiling System kit assays were performed at least in two individual experiments, each having two replicates.

#### Testing predicted targets for a kinase inhibitor tivozanib

For tivozanib’s off-target testing, ABL1, RPS6KB, SLK, Aurora A, HIPK4, FYN A, FRK, as well as their substrates and buffers were purchased from SignalChem (product codes and assay concentrations available in S6 Table). Kinase assay, kinase dilution and substrate buffers were as indicated by the manufacturer (Promega). The assays were performed in the same way as with the kinases from the Kinase Selectivity Profiling System + ADP-Glo Assay kits (see previous section). All the assays were done in three individual experiments, each having three replicates.

#### Analysis of the kinase assay results

IC_50_ values from all the experiments were obtained with GraphPad Prism 7 (GraphPad Software, Inc. California, USA) using *inhibitor vs*. *response—variable slope (four parameters)* analysis. Constrain bottom was set to *must be between zero and 100*, and constrain hill slope was set to *absolute value must be less than 1*.*5*. X-axis scale was set to log_10_. Sigmoidal dose-response curves are shown in S14 and S15 Figs.

## Supporting information

S1 Fig **(a) Leave-one-out and (b) leave-drug-out cross-validation results.** The prediction accuracy was assessed with root mean squared error (RMSE) between binding affinities (pK_i_) from the study by Metz *et al*. and those predicted using KronRLS model with different pairs of drug (rows) and protein (columns) molecular descriptors encoded as kernel matrices (**c**). The lower the RMSE value, the more accurate the model prediction. Of note, Gaussian interaction profile drug kernel (KD-GIP) which resulted in the highest predictive performance under the *Bioactivity Imputation* scenario (a) has not been evaluated under the *New Drug* setup (b) because it is constructed based on the bioactivity profile of a drug, information that in practice is unavailable when predicting target interactions for a new investigational drug compound.
(PDF)

S2 Fig **(a,b) Scatter plots between compound-kinase binding affinities (pK_i_) measured in the Metz *et al*. study and their model predictions under the (a) *Bioactivity Imputation*, (b) *New Drug* setups, using KronRLS algorithm with the best pairs of drug and protein kernels.** r indicates Pearson correlation and p-values were calculated using a Student's t distribution for a transformation of the correlation, as implemented in MATLAB Statistics Toolbox. Each point corresponds to one of 16,265 compound-kinase pairs. Most of the assays were performed at the inhibitor concentrations of 3–10,000 nM (corresponding to minimum pK_i_ of 5 M); however, some affinities were larger than 10,000 nM explaining the few outlier points with pK_i_ < 5 M. The higher the pK_i_ value, the stronger the affinity between the two molecules. Red lines mark a relatively stringent interaction threshold (7 M), distinguishing the top left corner as the region containing false positive interaction predictions, and the bottom right corner as false negative predictions. **(c,d) Receiver operating characteristic (ROC) curves constructed under the (c) *Bioactivity Imputation* and (d) *New Drug* setups using 21 different interaction threshold values (pK_i_ varying between 6 and 8 M with a step of 0.1 M) to binarize binding affinities measured in the Metz *et al*. study into true class labels.** Multiple values were used to study the ability of the model to discriminate interacting from non-interacting compound-kinase pairs at various interaction thresholds. The curves corresponding to the threshold of pK_i_ = 7 M, marked at the scatter plots (a) and (b), are plotted with the darkest red colour. AUC indicates the area under the ROC curve; the closer AUC is to 1, the more accurate the model prediction.
(PDF)

S3 Fig Distribution of 152 drug-wise Pearson correlation values between compound-kinase binding affinities (pK_i_) measured in the Metz *et al*. study and their model predictions under the *New Drug* scenario. The predictions were made using KronRLS algorithm with the best pair of drug and protein kernels (KD-sp and KP-GS) under the leave-drug-out cross-validation. 125 out of 152 correlations are statistically significant (p < 0.0001), and 58 correlation values are greater than 0.75.
(PDF)

S4 Fig Scatter plots between the measured compound-kinase binding affinities from Metz *et al*. study and their model predictions obtained using KronRLS with KD-GIP drug kernel and KP-GS-domain protein kernel. r indicates Pearson correlation and p-values were calculated using a Student's t distribution for a transformation of the correlation, as implemented in MATLAB Statistics Toolbox. Each plot corresponds to a single compound selected for the experimental validation, and each point represents its bioactivity against a single kinase. Points marked with red stars and orange dots are predictions for the unmeasured in Metz *et al*. study compound-kinase pairs; thus, they are placed on the y-axis. Red star-shapes and green triangular points indicate interactions included in our experimental validation. However, the green points constitute merely an experimental control as those binding affinities are reported in Metz *et al*. data set. The higher the pK_i_ value, the stronger the affinity between the two molecules. Red lines mark a pre-defined interaction threshold (7 M), distinguishing top left corner as the region containing false positive (FPs) interaction predictions, and bottom right corner as false negative (FN) predictions. During the selection of the molecules for the experimental validation, we aimed at minimizing the number of FPs and FNs. For instance, in case of cediranib (a), there are no FPs, meaning that the predicted interaction, indicated by the red star, is likely a true one. The percentage of missing values indicates unmeasured binding affinities of a given compound against a set of 138 kinases in the Metz *et al*. data set.
(PDF)

S5 Fig The comparison between model-predicted and experimentally-measured in different assays bioactivities of 100 compound-kinase pairs included in our experimental validation. **(a,c)** Technical variability between two experimental kinase assays. Scatter plots between **(a)** 82 pK_i_ values measured in Metz *et al*. study and pIC_50_ values from our experimental assay; **(c)** 95 pK_d_ values from Davis *et al*. study and pIC_50_ values from our experimental assay; **(b)** 73 *in silico*-predicted and measured in Metz *et al*. study pK_i_ values, excluding compound-kinase pairs blinded in the model training (marked with orange colour in Fig 4A and S2 Table); **(d)** 95 *in silico*-predicted pK_i_ values and pK_d_ readouts from Davis *et al*. study. The values are detailed in S2 Table.
(PDF)

S6 Fig The comparison between model-predicted (based on the data from Metz *et al*. study) and experimentally-measured (in the study by Davis *et al*.) compound-kinase bioactivities. **(a)** The *Bioactivity Imputation* scenario. The KronRLS model was trained using *Metz et al*. dataset together with the best-performing (under the *Bioactivity Imputation* scenario) drug interaction profile kernel (KD-GIP) and kinase domain-based generic string protein kernel (KP-GS-domain, Fig 3A). The model was then used to predict binding affinities between 2,662 compound-kinase pairs overlapping between Metz *et al*. and Davis *et al*. datasets. **(b)** The *New Target* scenario. The KronRLS algorithm was trained using *Metz et al*. dataset together with the best-performing (under the *New Target* scenario) PubChem’s fingerprint-based drug kernel (KD-PubChem-2D) and extended target profile protein kernel built upon Smith-Waterman amino acid sequence comparisons (KP-SW+, S7 Fig). The model was then used to predict the binding affinities between 152 drugs from Metz *et al*. study and 244 wild-type kinases present in Davis *et al*. but not Metz *et al*. dataset. The predictive performance was evaluated based on 5,368 binding affinities of 22 drugs overlapping between the two datasets.
(PDF)

S7 Fig **(a) Leave-target-out cross-validation results.** The prediction accuracy was evaluated with Pearson correlation (r) between binding affinities (pK_i_) from the study by Metz *et al*. and those predicted using KronRLS algorithm with different pairs of drug (rows) and protein (columns) molecular descriptors encoded as kernel matrices (b).
(PDF)

S8 Fig Kinase dendrogram created by hierarchical clustering of 138 kinases from Metz *et al*. study based on their bioactivity data (pK_i_). The bar length is proportional to the number of compound interactions for each kinase (pK_i_ ≥ 7 M). On-targets of tivozanib are marked with red lines (*FLT1*, *FLT4*, *KDR*), fedratinib–green lines (*JAK2*, *JAK3*, *TYK2*), vx11e and ulixertinib (a compound derived from vx11e)–blue lines (vx11e: *CDK2*, *ERK2*; ulixertinib: *ERK2*).
(PDF)

S9 Fig Interaction map between 152 compounds (rows) and 138 kinases (columns) profiled in the study of Metz *et al*. The white cells represent unmeasured binding affinities. The higher the pK_i_ value, the stronger the affinity between the compound and kinase.
(PDF)

S10 Fig Distribution of 16,265 compound-kinase binding affinities measured in the study of Metz *et al*. (PDF)

S11 Fig Kinome map of 138 kinases used in our work. Figure was created with KinMap (http://kinhub.org/kinmap).
(PDF)

S12 Fig Schematic illustration of the nested leave-one-out cross-validation (LOO-CV) procedure, consisting of the inner loop for model selection, and the outer loop for model performance estimation. Single round of the outer CV is shown, where one compound-protein pair is removed from the training data, and used as a test fold. The inner leave-one-out CV is run for each model parameter combination (grid search) during every round of the outer CV. The combination resulting in the lowest root mean squared error between the original and predicted binding affinities is selected and used in the model training in the outer CV loop. We used LOO-CV to tune the model parameters and assess its performance under the *Bioactivity Imputation* scenario (Fig 2A).
(PDF)

S13 Fig Schematic illustration of the nested leave-drug-out cross-validation (LDO-CV) procedure consisting of the inner loop for model selection, and the outer loop for model performance estimation. Single round of the outer CV is shown, where all binding affinities of selected compound are removed from the training data, and used as a test fold. The inner leave-drug-out CV is run for each model parameter combination (grid search) during every round of the outer CV. In the inner CV, five compounds are removed at a time (at random). The model parameter combination resulting in the lowest root mean squared error between the original and predicted binding affinities is selected and used in the model training in the outer CV loop. Note that also the rows and columns corresponding to the compounds included in the test fold are removed from the drug kernel matrix before model training. We used LDO-CV to tune the model parameters and assess its performance under the *New Drug* scenario (Fig 2B).
(PDF)

S14 Fig Results of our kinase assay for testing bioactivities predicted to fill the experimental gaps in the large-scale kinase inhibitor target profiling study by Metz *et al*.; examples of drug response curves obtained as described in Materials and Methods section of the main paper. Corresponding pIC_50_ values are summarized in S2 Table.
(PDF)

S15 Fig Results of our kinase assay for testing predicted target interactions for a new investigational kinase inhibitor tivozanib; drug response curves obtained as described in Materials and Methods section of the main paper. Corresponding pIC_50_ values are summarized in S3 Table.
(PDF)

S16 Fig Visualisation of the binding between tivozanib and *ABL1* (PDB code: 2e2b). The docking was performed with Rosetta (https://www.rosettacommons.org/) and the figure was created using UCSF cHimera (https://www.cgl.ucsf.edu/chimera/). A radius for docking was set to 5 Å around the centre of the ATP-binding site.
(PNG)

S1 Table Possible drug-protein interaction prediction scenarios. In this work, we focused on two most practical ones, namely the *Bioactivity Imputation* (Fig 2A) and the *New Drug* (Fig 2B) scenarios. Additionally, we included the results under the *New Target* setup (Fig 2C) in S7 Fig. (*d*_*x*_, *p*_*x*_) denotes the query drug-protein pair, the binding affinity of which one aims to predict.
(PDF)

S2 Table Results of the experimental validation of bioactivities predicted to fill the gaps in the study of Metz *et al*. (XLSX)

S3 Table Predicted target profile of tivozanib and the results of experimental validation. (XLSX)

S4 Table Model parameters tested. In case of Gaussian kernels (**K**_*D-GIP*_, **K**_*P-GIP*_), the values for kernel width parameter *σ* were selected by computing pairwise distances between all data points and taking 0.1, 0.5 and 0.9 quantiles.
(PDF)

S5 Table Kinase inhibitors used in our experimental assays. (PDF)

S6 Table Kinases, substrates, and their concentrations used in tivozanib’s off-target testing. (PDF)
