# Supplementary material for: Computational-experimental approach to drug-target interaction mapping: A case study on kinase inhibitors
Source: PLoS Comput Biol. 2017 Aug 7;13(8):e1005678. doi: 10.1371/journal.pcbi.1005678 (PMC5560747; doi:10.1371/journal.pcbi.1005678)
Supplement: S2 Fig — (a,b) Scatter plots between compound-kinase binding affinities (pKi) measured in the Metz et al. study and their model predictions under the (a) Bioactivity Imputation, (b) New Drug setups, using KronRLS algorithm with the best pairs of drug and protein kernels. r indicates Pearson correlation and p-values were calculated using a Student's t distribution for a transformation of the correlation, as implemented in MATLAB Statistics Toolbox. Each point corresponds to one of 16,265 compound-kinase pairs. Most of the assays were performed at the inhibitor concentrations of 3–10,000 nM (corresponding to minimum pKi of 5 M); however, some affinities were larger than 10,000 nM explaining the few outlier points with pKi < 5 M. The higher the pKi value, the stronger the affinity between the two molecules. Red lines mark a relatively stringent interaction threshold (7 M), distinguishing the top left corner as the region containing false positive interaction predictions, and the bottom right corner as false negative predictions. (c,d) Receiver operating characteristic (ROC) curves constructed under the (c) Bioactivity Imputation and (d) New Drug setups using 21 different interaction threshold values (pKi varying between 6 and 8 M with a step of 0.1 M) to binarize binding affinities measured in the Metz et al. study into true class labels. Multiple values were used to study the ability of the model to discriminate interacting from non-interacting compound-kinase pairs at various interaction thresholds. The curves corresponding to the threshold of pKi = 7 M, marked at the scatter plots (a) and (b), are plotted with the darkest red colour. AUC indicates the area under the ROC curve; the closer AUC is to 1, the more accurate the model prediction. (PDF) [file pcbi.1005678.s002.pdf]

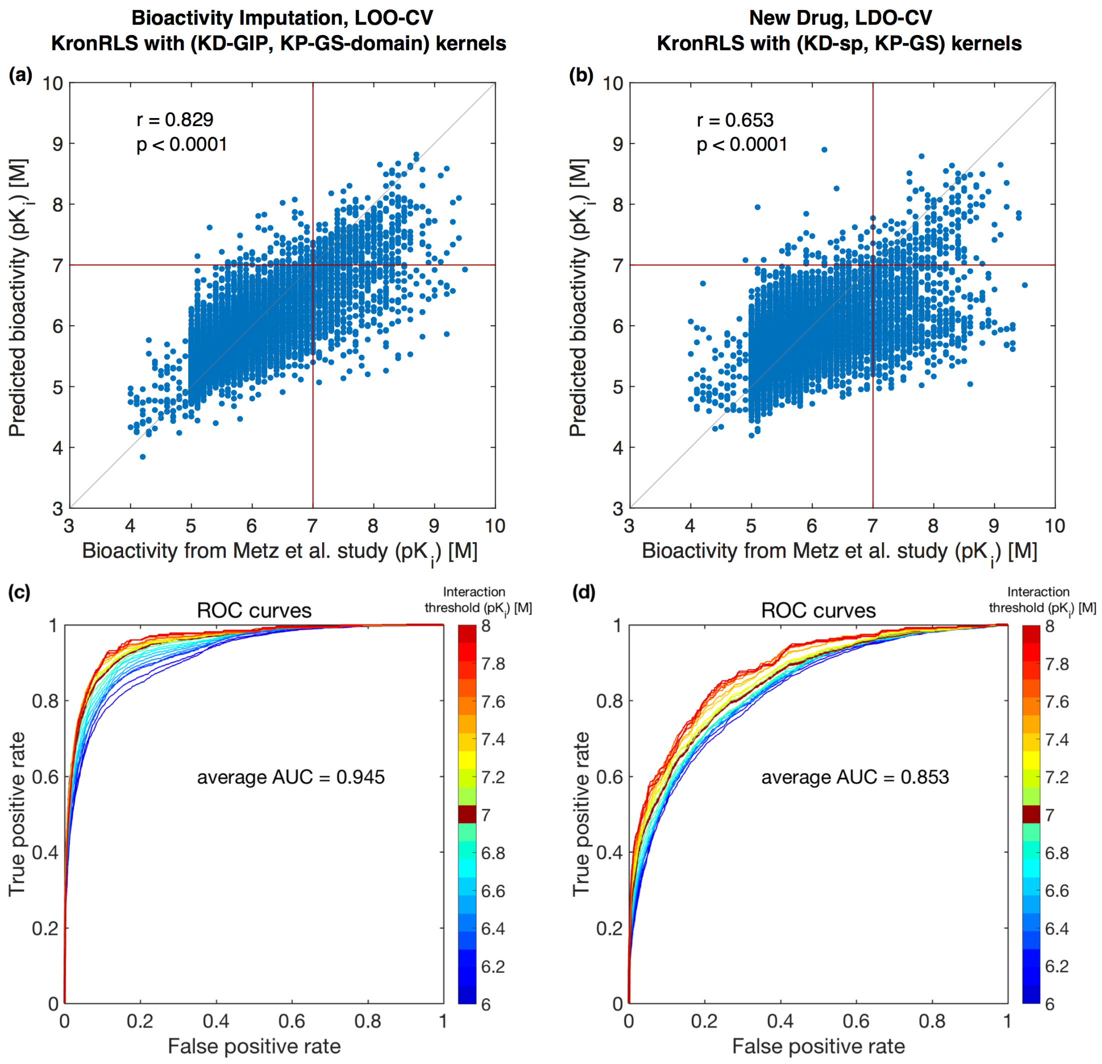


**S2 Fig. (a,b) Scatter plots between compound-kinase binding affinities ($pK_i$) measured in the Metz *et al.* study and their model predictions under the (a) *Bioactivity Imputation*, (b) *New Drug* setups, using KronRLS algorithm with the best pairs of drug and protein kernels.** r indicates Pearson correlation and p-values were calculated using a Student's t distribution for a transformation of the correlation, as implemented in MATLAB Statistics Toolbox. Each point corresponds to one of 16,265 compound-kinase pairs. Most of the assays were performed at the inhibitor concentrations of 3-10,000 nM (corresponding to minimum $pK_i$ of 5 M); however, some affinities were larger than 10,000 nM explaining the few outlier points with $pK_i < 5$ M. The higher the $pK_i$ value, the stronger the affinity between the two molecules. Red lines mark a relatively stringent interaction threshold (7 M), distinguishing the top left corner as the region containing false positive interaction predictions, and the bottom right corner as false negative predictions. **(c,d) Receiver operating characteristic (ROC) curves constructed under the (c) *Bioactivity Imputation* and (d) *New Drug* setups using 21 different interaction threshold values ($pK_i$ varying between 6 and 8 M with a step of 0.1 M) to binarize binding affinities measured in the Metz *et al.* study into true class labels.** Multiple values were used to study the ability of the model to discriminate interacting from non-interacting compound-kinase pairs at various interaction thresholds. The curves corresponding to the threshold of $pK_i$ = 7 M, marked at the scatter plots (a) and (b), are plotted with the darkest red colour. AUC indicates the area under the ROC curve; the closer AUC is to 1, the more accurate the model prediction.
