# Supplementary material for: Computational-experimental approach to drug-target interaction mapping: A case study on kinase inhibitors
Source: PLoS Comput Biol. 2017 Aug 7;13(8):e1005678. doi: 10.1371/journal.pcbi.1005678 (PMC5560747; doi:10.1371/journal.pcbi.1005678)
Supplement: S3 Fig — The predictions were made using KronRLS algorithm with the best pair of drug and protein kernels (KD-sp and KP-GS) under the leave-drug-out cross-validation. 125 out of 152 correlations are statistically significant (p < 0.0001), and 58 correlation values are greater than 0.75. (PDF) [file pcbi.1005678.s003.pdf]

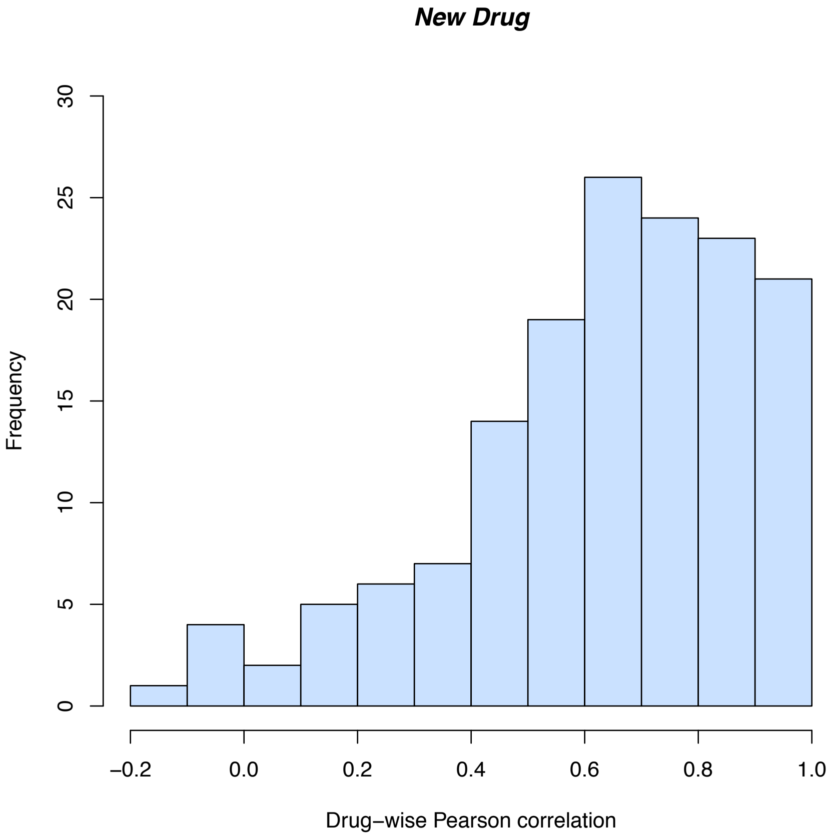

S3 Fig. Distribution of 152 drug-wise Pearson correlation values between compound-kinase binding affinities (pK_i) measured in the Metz *et al.* study and their model predictions under the *New Drug* scenario. The predictions were made using KronRLS algorithm with the best pair of drug and protein kernels (KD-sp and KP-GS) under the leave-drug-out cross-validation. 125 out of 152 correlations are statistically significant ($p < 0.0001$), and 58 correlation values are greater than 0.75.
