# Supplementary material for: Computational-experimental approach to drug-target interaction mapping: A case study on kinase inhibitors
Source: PLoS Comput Biol. 2017 Aug 7;13(8):e1005678. doi: 10.1371/journal.pcbi.1005678 (PMC5560747; doi:10.1371/journal.pcbi.1005678)
Supplement: S4 Fig — r indicates Pearson correlation and p-values were calculated using a Student's t distribution for a transformation of the correlation, as implemented in MATLAB Statistics Toolbox. Each plot corresponds to a single compound selected for the experimental validation, and each point represents its bioactivity against a single kinase. Points marked with red stars and orange dots are predictions for the unmeasured in Metz et al. study compound-kinase pairs; thus, they are placed on the y-axis. Red star-shapes and green triangular points indicate interactions included in our experimental validation. However, the green points constitute merely an experimental control as those binding affinities are reported in Metz et al. data set. The higher the pKi value, the stronger the affinity between the two molecules. Red lines mark a pre-defined interaction threshold (7 M), distinguishing top left corner as the region containing false positive (FPs) interaction predictions, and bottom right corner as false negative (FN) predictions. During the selection of the molecules for the experimental validation, we aimed at minimizing the number of FPs and FNs. For instance, in case of cediranib (a), there are no FPs, meaning that the predicted interaction, indicated by the red star, is likely a true one. The percentage of missing values indicates unmeasured binding affinities of a given compound against a set of 138 kinases in the Metz et al. data set. (PDF) [file pcbi.1005678.s004.pdf]

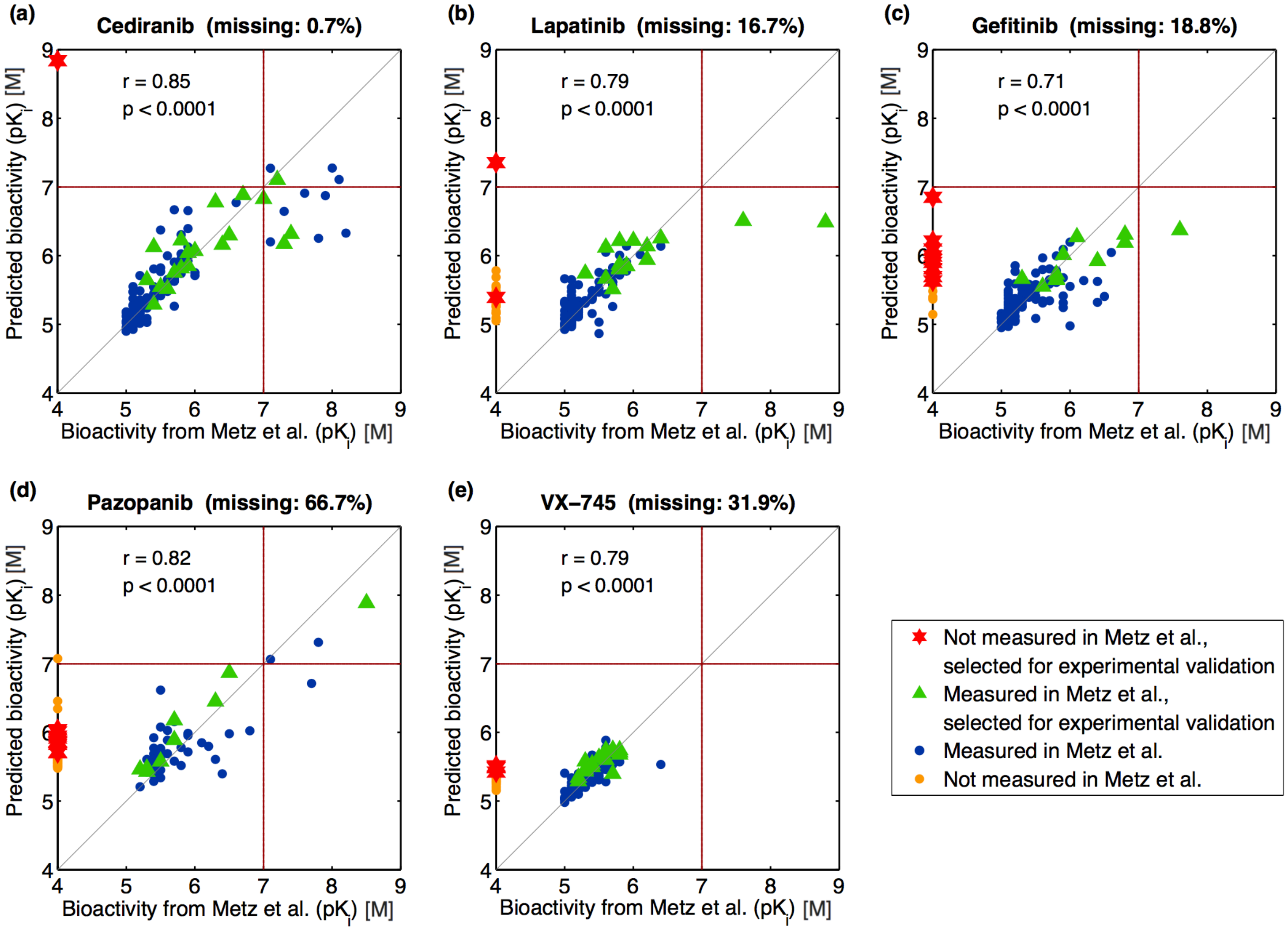

S4 Fig. Scatter plots between the measured compound-kinase binding affinities from Metz *et al.* study and their model predictions obtained using KronRLS with KD-GIP drug kernel and KP-GS-domain protein kernel. r indicates Pearson correlation and p-values were calculated using a Student's t distribution for a transformation of the correlation, as implemented in MATLAB Statistics Toolbox. Each plot corresponds to a single compound selected for the experimental validation, and each point represents its bioactivity against a single kinase. Points marked with red stars and orange dots are predictions for the unmeasured in Metz *et al*. study compound-kinase pairs; thus, they are placed on the y-axis. Red star-shapes and green triangular points indicate interactions included in our experimental validation. However, the green points constitute merely an experimental control as those binding affinities are reported in Metz *et al*. data set. The higher the pK_i value, the stronger the affinity between the two molecules. Red lines mark a pre-defined interaction threshold (7 M), distinguishing top left corner as the region containing false positive (FPs) interaction predictions, and bottom right corner as false negative (FN) predictions. During the selection of the molecules for the experimental validation, we aimed at minimizing the number of FPs and FNs. For instance, in case of cediranib (a), there are no FPs, meaning that the predicted interaction, indicated by the red star, is likely a true one. The percentage of missing values indicates unmeasured binding affinities of a given compound against a set of 138 kinases in the Metz *et al*. data set.
