# Supplementary material for: Computational-experimental approach to drug-target interaction mapping: A case study on kinase inhibitors
Source: PLoS Comput Biol. 2017 Aug 7;13(8):e1005678. doi: 10.1371/journal.pcbi.1005678 (PMC5560747; doi:10.1371/journal.pcbi.1005678)
Supplement: S5 Fig — (a,c) Technical variability between two experimental kinase assays. Scatter plots between (a) 82 pKi values measured in Metz et al. study and pIC50 values from our experimental assay; (c) 95 pKd values from Davis et al. study and pIC50 values from our experimental assay; (b) 73 in silico-predicted and measured in Metz et al. study pKi values, excluding compound-kinase pairs blinded in the model training (marked with orange colour in Fig 4A and S2 Table); (d) 95 in silico-predicted pKi values and pKd readouts from Davis et al. study. The values are detailed in S2 Table. (PDF) [file pcbi.1005678.s005.pdf]

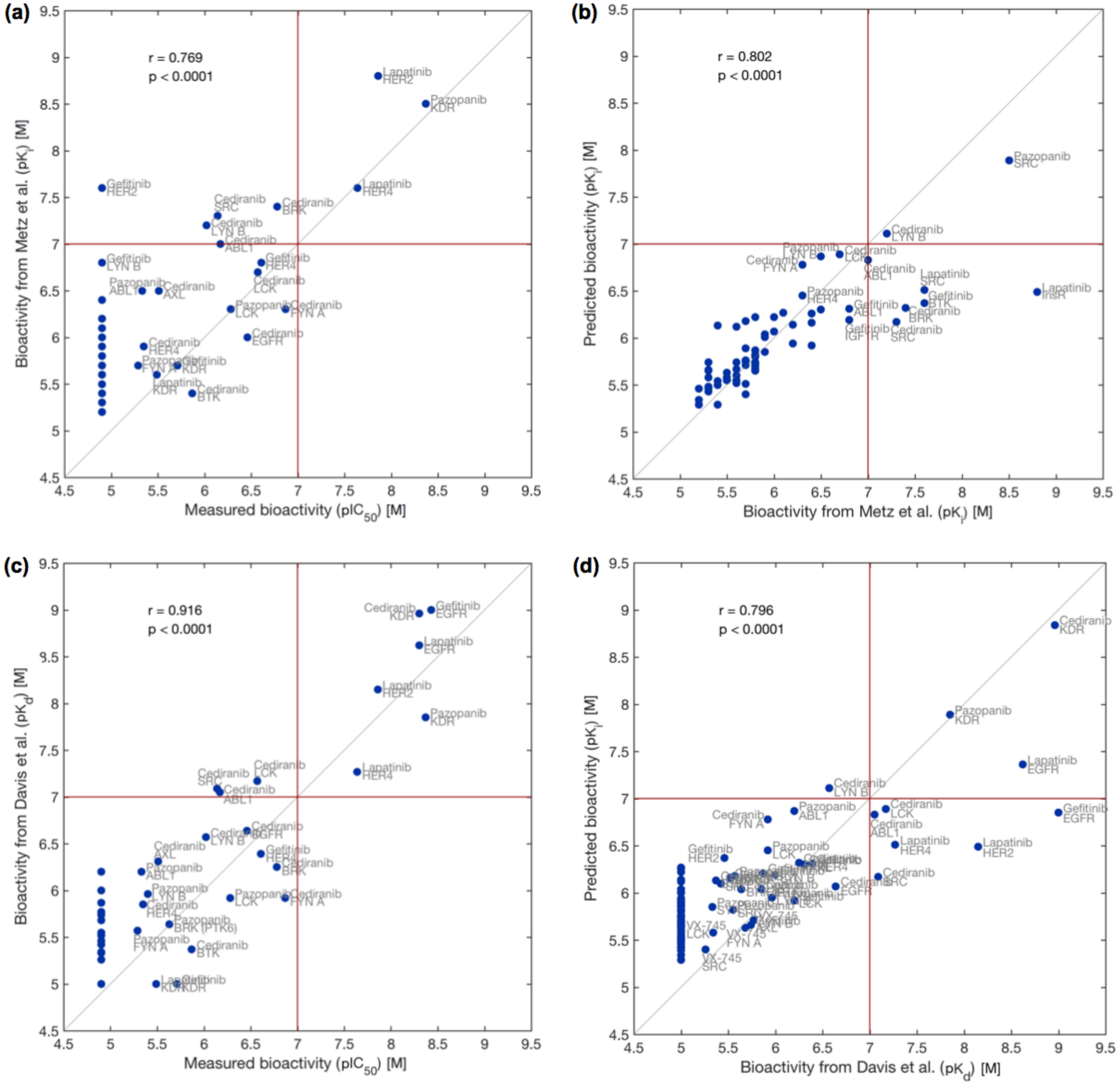

S5 Fig. The comparison between model-predicted and experimentally-measured in different assays bioactivities of 100 compound-kinase pairs included in our experimental validation. **(a,c)** Technical variability between two experimental kinase assays. Scatter plots between **(a)** 82 pK_i values measured in Metz *et al*. study and pIC_{50} values from our experimental assay; **(c)** 95 pK_d values from Davis *et al*. study and pIC_{50} values from our experimental assay; **(b)** 73 *in silico*-predicted and measured in Metz *et al*. study pK_i values, excluding compound-kinase pairs blinded in the model training (marked with orange colour in Fig 4a and S2 Table); **(d)** 95 *in silico*-predicted pK_i values and pK_d readouts from Davis *et al*. study. The values are detailed in S2 Table.
