# Supplementary material for: Computational-experimental approach to drug-target interaction mapping: A case study on kinase inhibitors
Source: PLoS Comput Biol. 2017 Aug 7;13(8):e1005678. doi: 10.1371/journal.pcbi.1005678 (PMC5560747; doi:10.1371/journal.pcbi.1005678)
Supplement: S6 Fig — (a) The Bioactivity Imputation scenario. The KronRLS model was trained using Metz et al. dataset together with the best-performing (under the Bioactivity Imputation scenario) drug interaction profile kernel (KD-GIP) and kinase domain-based generic string protein kernel (KP-GS-domain, Fig 3A). The model was then used to predict binding affinities between 2,662 compound-kinase pairs overlapping between Metz et al. and Davis et al. datasets. (b) The New Target scenario. The KronRLS algorithm was trained using Metz et al. dataset together with the best-performing (under the New Target scenario) PubChem’s fingerprint-based drug kernel (KD-PubChem-2D) and extended target profile protein kernel built upon Smith-Waterman amino acid sequence comparisons (KP-SW+, S7 Fig). The model was then used to predict the binding affinities between 152 drugs from Metz et al. study and 244 wild-type kinases present in Davis et al. but not Metz et al. dataset. The predictive performance was evaluated based on 5,368 binding affinities of 22 drugs overlapping between the two datasets. (PDF) [file pcbi.1005678.s006.pdf]

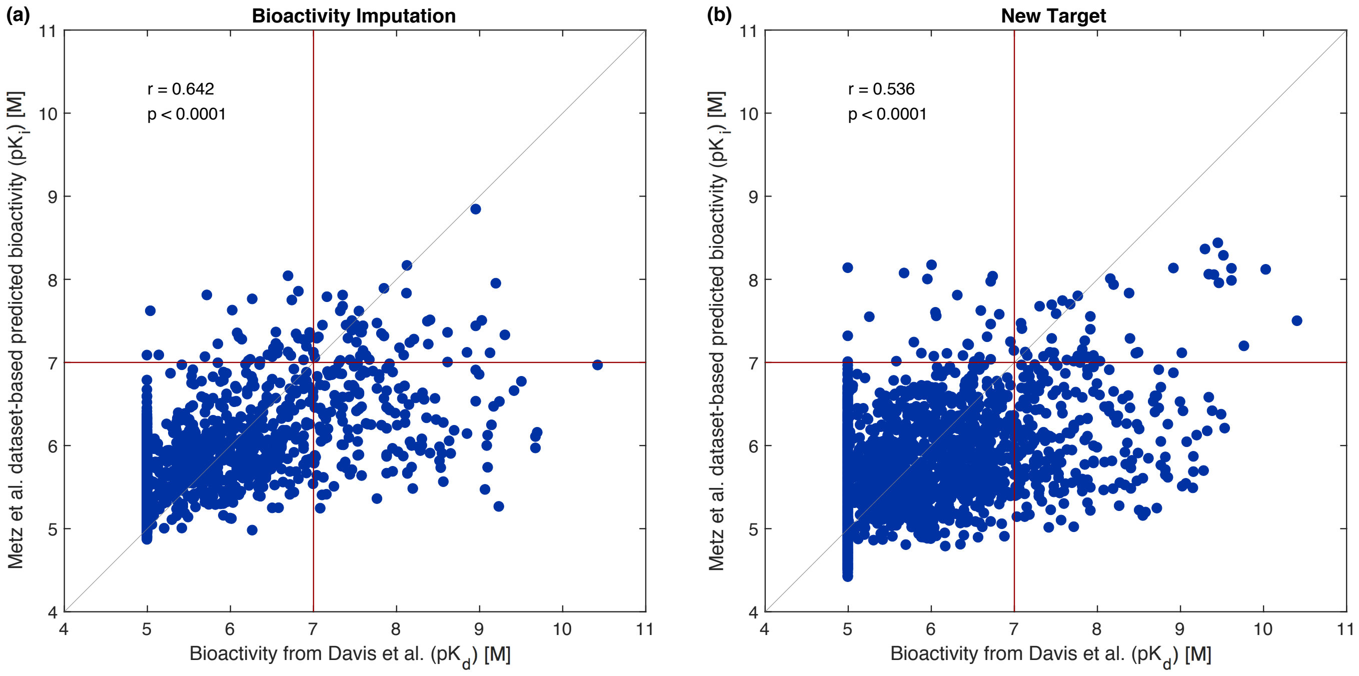

S6 Fig. The comparison between model-predicted (based on the data from Metz *et al.* study) and experimentally-measured (in the study by Davis *et al.*) compound-kinase bioactivities. (a) The *Bioactivity Imputation* scenario. The KronRLS model was trained using Metz *et al*. dataset together with the best-performing (under the *Bioactivity Imputation* scenario) drug interaction profile kernel (KD-GIP) and kinase domain-based generic string protein kernel (KP-GS-domain, Fig 3a). The model was then used to predict binding affinities between 2,662 compound-kinase pairs overlapping between Metz *et al*. and Davis *et al*. datasets. **(b)** The *New Target* scenario. The KronRLS algorithm was trained using Metz *et al*. dataset together with the best-performing (under the *New Target* scenario) PubChem's fingerprint-based drug kernel (KD-PubChem-2D) and extended target profile protein kernel built upon Smith-Waterman amino acid sequence comparisons (KP-SW+, S7 Fig). The model was then used to predict the binding affinities between 152 drugs from Metz *et al*. study and 244 wild-type kinases present in Davis *et al*. but not Metz *et al*. dataset. The predictive performance was evaluated based on 5,368 binding affinities of 22 drugs overlapping between the two datasets.
