# Supplementary material for: Computational-experimental approach to drug-target interaction mapping: A case study on kinase inhibitors
Source: PLoS Comput Biol. 2017 Aug 7;13(8):e1005678. doi: 10.1371/journal.pcbi.1005678 (PMC5560747; doi:10.1371/journal.pcbi.1005678)
Supplement: S7 Fig — (a) Leave-target-out cross-validation results. The prediction accuracy was evaluated with Pearson correlation (r) between binding affinities (pKi) from the study by Metz et al. and those predicted using KronRLS algorithm with different pairs of drug (rows) and protein (columns) molecular descriptors encoded as kernel matrices (b). (PDF) [file pcbi.1005678.s007.pdf]

(a)

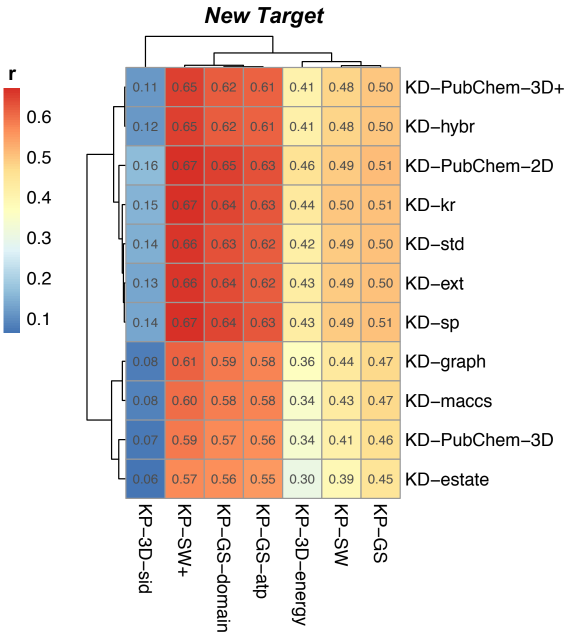

(b)

	Abbreviation	Description	
Drug compounds	KD-PubChem-2D	881-bit fingerprint based on 2D substructures defined by PubChem	Fingerprint-based linear kernels
	KD-PubChem-3D	PubChem's descriptor based on up to 10 calculated 3D conformers	
	KD-PubChem-3D+	PubChem's descriptor based on up to 10 calculated 3D conformers, additionally including information on acceptor count, hydrophobe count and ring count	
	KD-estate	79-bit fingerprint corresponding to EState substructures	
	KD-ext	1024-bit path-based, hashed fingerprint taking into account ring systems	
	KD-graph	1024-bit path-based, hashed fingerprint considering connectivity	
	KD-hybr	1024-bit path-based, hashed fingerprint considering hybridization states	
	KD-kr	4860-bit fingerprint defined by Klekota and Roth	
	KD-maccs	166-bit fingerprint based on MACCS structural keys	
	KD-sp	1024-bit fingerprint based on the shortest paths between pairs of atoms taking into account ring systems and charges	
	KD-std	1024-bit path-based, hashed fingerprint	
Protein targets	KP-SW	Amino acid sequence-based linear kernel	
	KP-SW+	Amino acid sequence and extended target profile-based linear kernel	
	KP-3D-energy	Protein structure-based linear kernel (using absolute value of the energy needed to align proteins)	
	KP-3D-sid	Protein structure-based linear kernel (using sequence identity)	
	KP-GS	Generic String kernel applied to full protein sequences	
	KP-GS-domain	Generic String kernel applied to kinase domain sequences	
	KP-GS-atp	Generic String kernel applied to ATP binding pocket sequences	

S7 Fig. (a) Leave-target-out cross-validation results. The prediction accuracy was evaluated with Pearson correlation (r) between binding affinities (pK_i) from the study by Metz *et al*. and those predicted using KronRLS algorithm with different pairs of drug (rows) and protein (columns) molecular descriptors encoded as kernel matrices **(b)**.
