# Supplementary material for: Computational-experimental approach to drug-target interaction mapping: A case study on kinase inhibitors
Source: PLoS Comput Biol. 2017 Aug 7;13(8):e1005678. doi: 10.1371/journal.pcbi.1005678 (PMC5560747; doi:10.1371/journal.pcbi.1005678)
Supplement: S9 Fig — The white cells represent unmeasured binding affinities. The higher the pKi value, the stronger the affinity between the compound and kinase. (PDF) [file pcbi.1005678.s009.pdf]

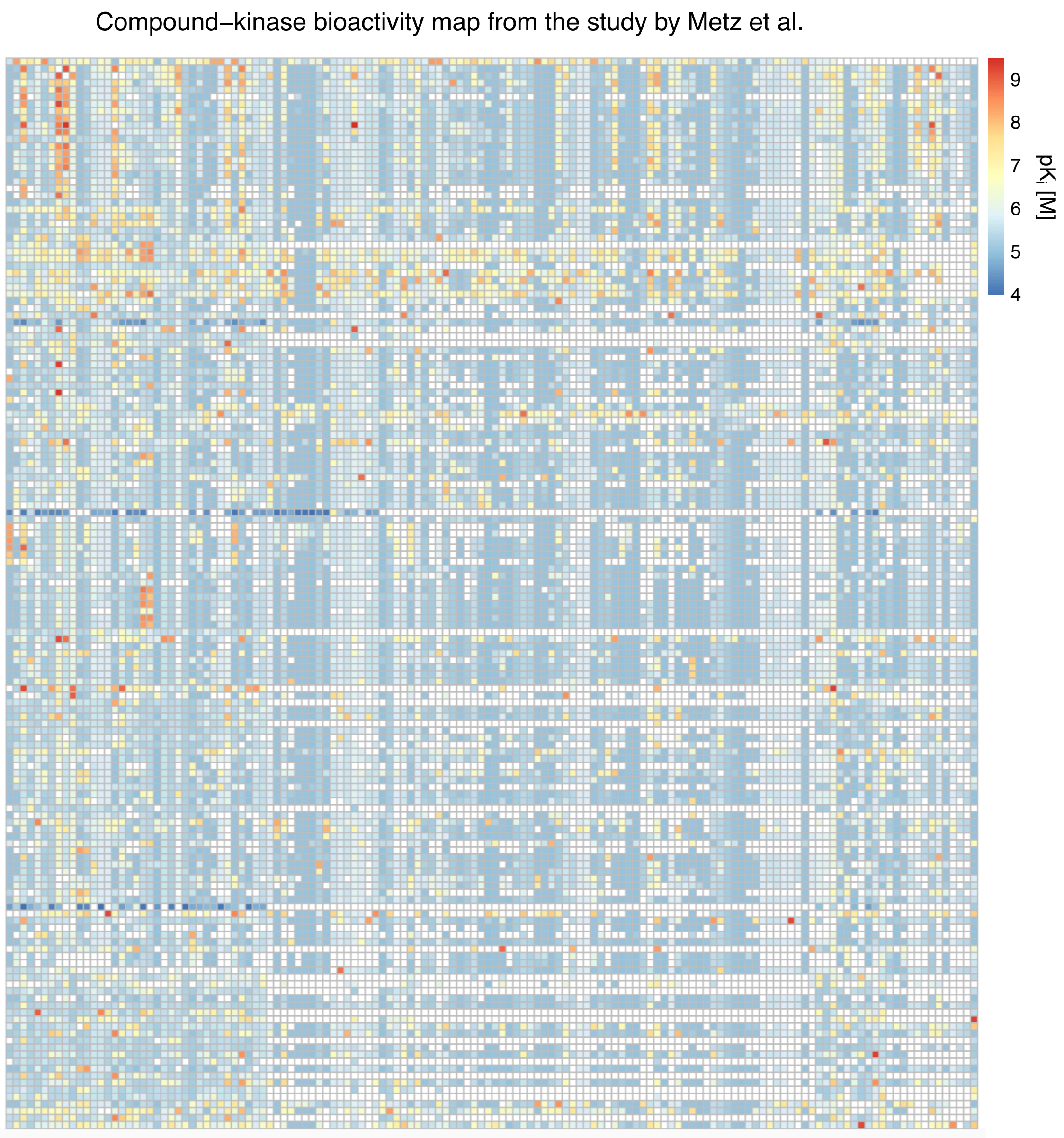

S9 Fig. Interaction map between 152 compounds (rows) and 138 kinases (columns) profiled in the study of Metz *et al.* The white cells represent unmeasured binding affinities. The higher the pK_i value, the stronger the affinity between the compound and kinase.
