# Supplementary figures and images for: Computational-experimental approach to drug-target interaction mapping: A case study on kinase inhibitors
Source: PLoS Comput Biol. 2017 Aug 7;13(8):e1005678. doi: 10.1371/journal.pcbi.1005678 (PMC5560747; doi:10.1371/journal.pcbi.1005678)

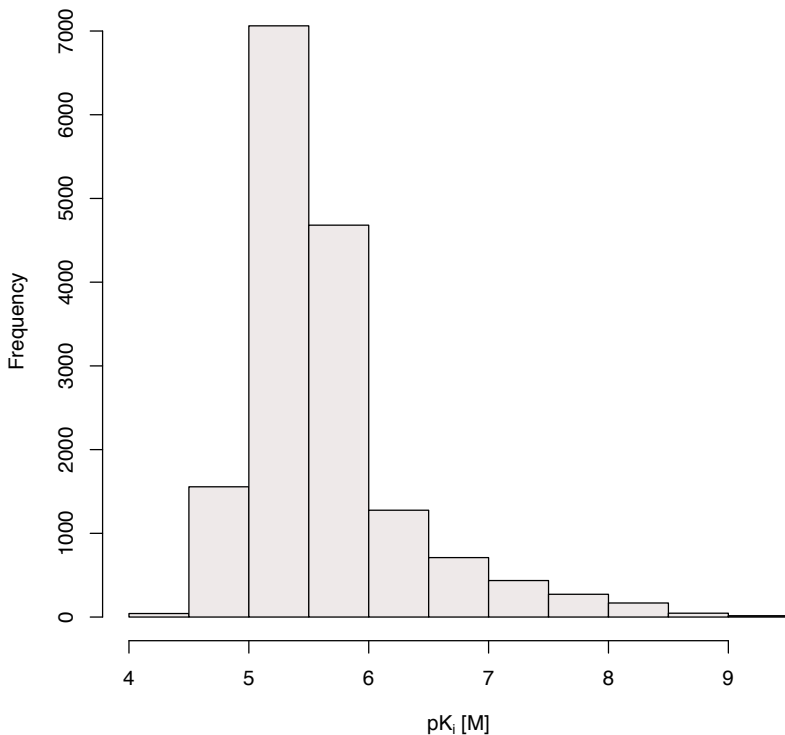

**S10 Fig. Distribution of 16,265 compound-kinase binding affinities measured in the study of Metz *et al.***

Supplement: S10 Fig — (PDF) [file pcbi.1005678.s010.pdf]

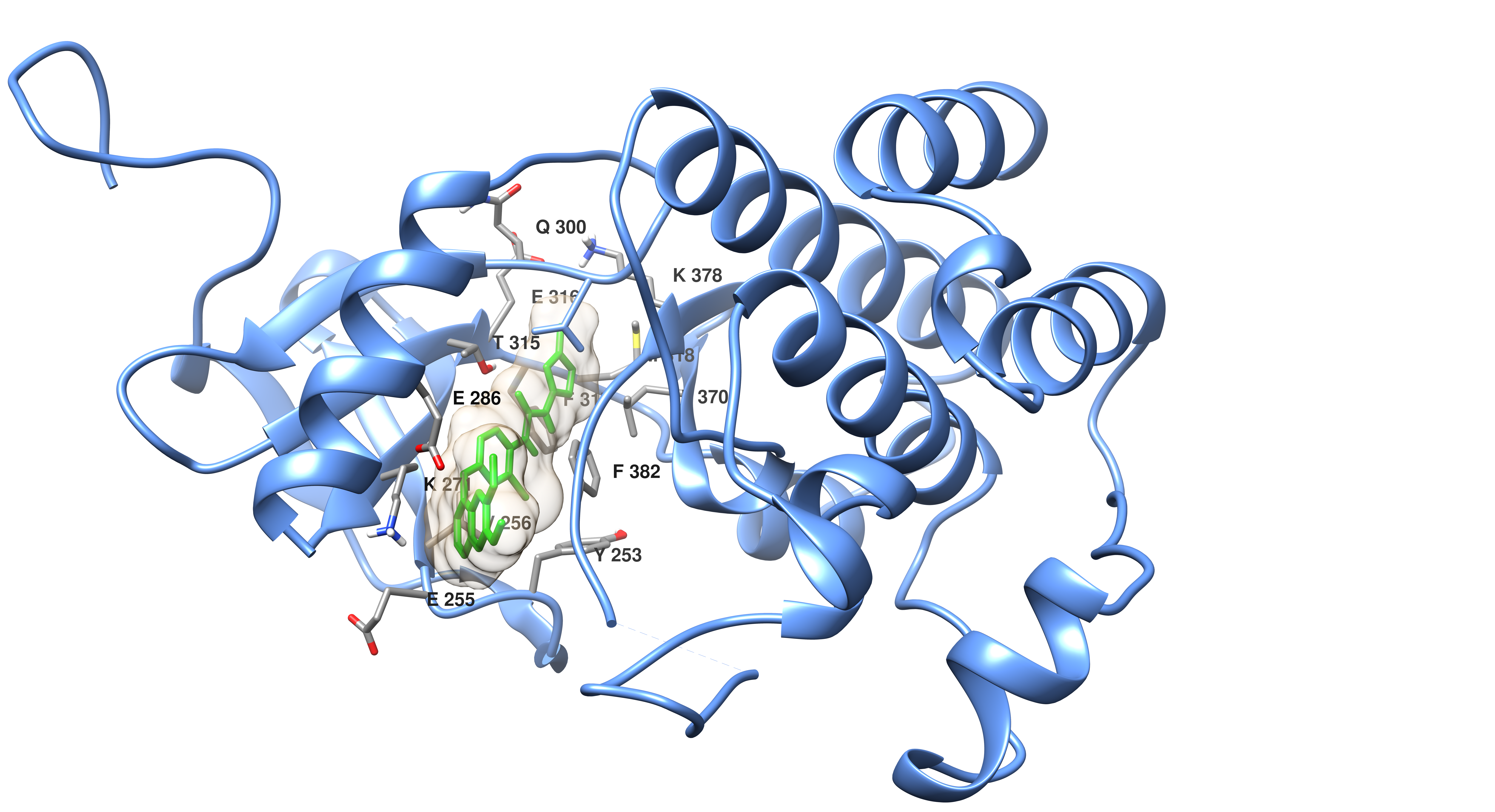

Supplement: S16 Fig — The docking was performed with Rosetta (https://www.rosettacommons.org/) and the figure was created using UCSF cHimera (https://www.cgl.ucsf.edu/chimera/). A radius for docking was set to 5 Å around the centre of the ATP-binding site. (PNG) [file pcbi.1005678.s016.png]
