# Supplementary material for: Computational-experimental approach to drug-target interaction mapping: A case study on kinase inhibitors
Source: PLoS Comput Biol. 2017 Aug 7;13(8):e1005678. doi: 10.1371/journal.pcbi.1005678 (PMC5560747; doi:10.1371/journal.pcbi.1005678)
Supplement: S11 Fig — Figure was created with KinMap (http://kinhub.org/kinmap). (PDF) [file pcbi.1005678.s011.pdf]

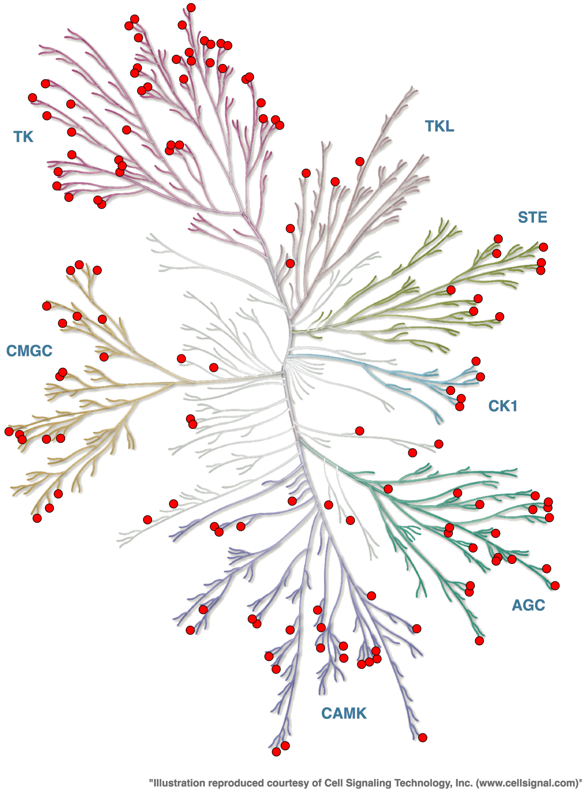

S11 Fig. Kinome map of 138 kinases used in our work.

Figure was created with KinMap (http://kinhub.org/kinmap).
