# Supplementary material for: Computational-experimental approach to drug-target interaction mapping: A case study on kinase inhibitors
Source: PLoS Comput Biol. 2017 Aug 7;13(8):e1005678. doi: 10.1371/journal.pcbi.1005678 (PMC5560747; doi:10.1371/journal.pcbi.1005678)
Supplement: S12 Fig — Single round of the outer CV is shown, where one compound-protein pair is removed from the training data, and used as a test fold. The inner leave-one-out CV is run for each model parameter combination (grid search) during every round of the outer CV. The combination resulting in the lowest root mean squared error between the original and predicted binding affinities is selected and used in the model training in the outer CV loop. We used LOO-CV to tune the model parameters and assess its performance under the Bioactivity Imputation scenario (Fig 2A). (PDF) [file pcbi.1005678.s012.pdf]

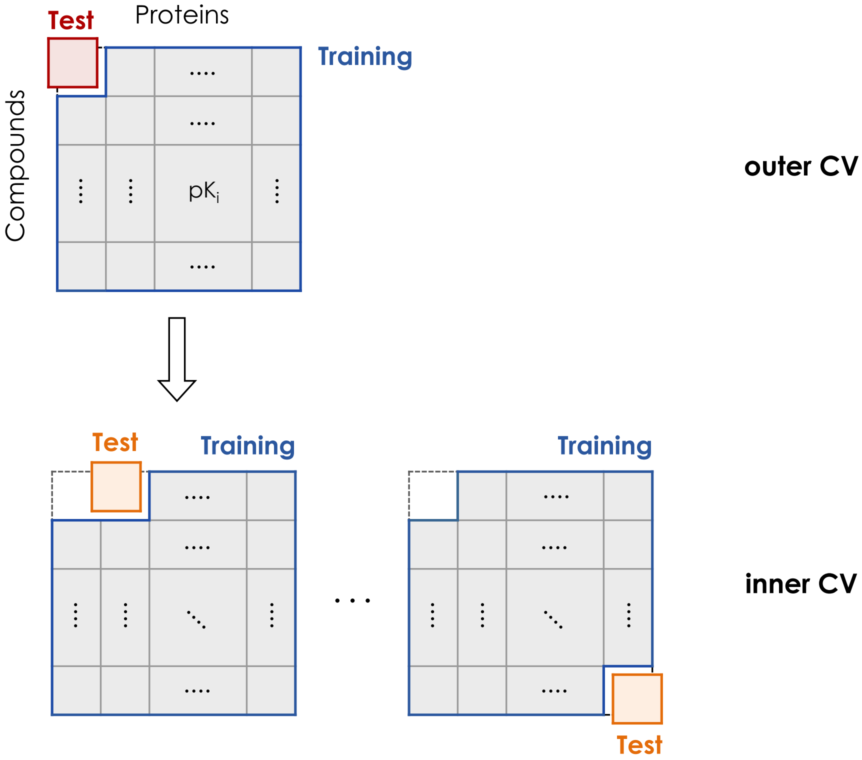

S12 Fig. Schematic illustration of the nested leave-one-out cross-validation (LOO-CV) procedure, consisting of the inner loop for model selection, and the outer loop for model performance estimation. Single round of the outer CV is shown, where one compound-protein pair is removed from the training data, and used as a test fold. The inner leave-one-out CV is run for each model parameter combination (grid search) during every round of the outer CV. The combination resulting in the lowest root mean squared error between the original and predicted binding affinities is selected and used in the model training in the outer CV loop. We used LOO-CV to tune the model parameters and assess its performance under the *Bioactivity Imputation* scenario (Fig 2a).
