# Supplementary material for: Computational-experimental approach to drug-target interaction mapping: A case study on kinase inhibitors
Source: PLoS Comput Biol. 2017 Aug 7;13(8):e1005678. doi: 10.1371/journal.pcbi.1005678 (PMC5560747; doi:10.1371/journal.pcbi.1005678)
Supplement: S13 Fig — Single round of the outer CV is shown, where all binding affinities of selected compound are removed from the training data, and used as a test fold. The inner leave-drug-out CV is run for each model parameter combination (grid search) during every round of the outer CV. In the inner CV, five compounds are removed at a time (at random). The model parameter combination resulting in the lowest root mean squared error between the original and predicted binding affinities is selected and used in the model training in the outer CV loop. Note that also the rows and columns corresponding to the compounds included in the test fold are removed from the drug kernel matrix before model training. We used LDO-CV to tune the model parameters and assess its performance under the New Drug scenario (Fig 2B). (PDF) [file pcbi.1005678.s013.pdf]

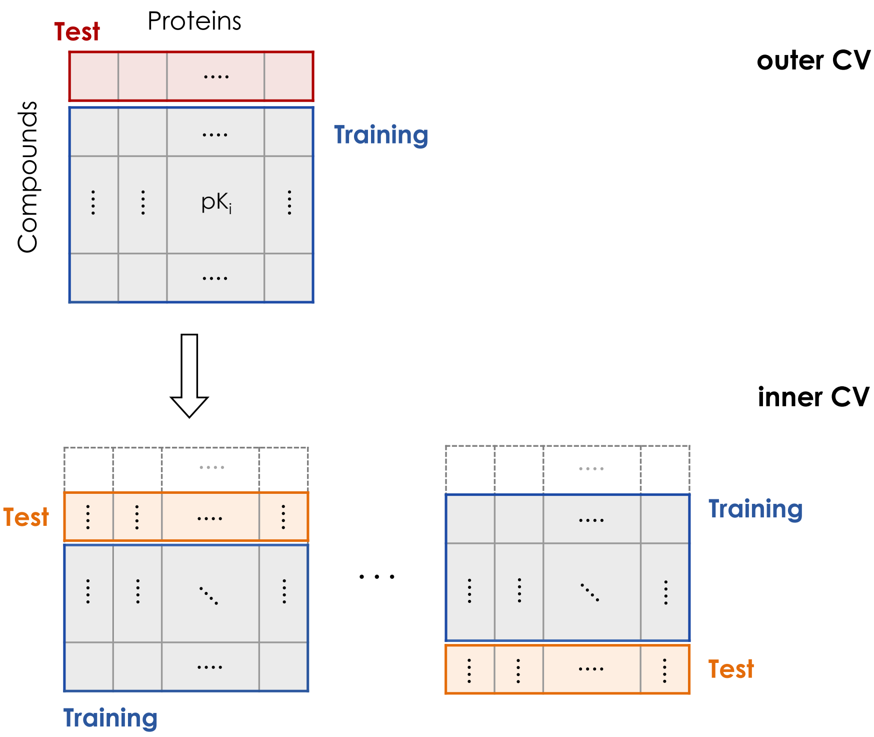

S13 Fig. Schematic illustration of the nested leave-drug-out cross-validation (LDO-CV) procedure consisting of the inner loop for model selection, and the outer loop for model performance estimation. Single round of the outer CV is shown, where all binding affinities of selected compound are removed from the training data, and used as a test fold. The inner leave-drug-out CV is run for each model parameter combination (grid search) during every round of the outer CV. In the inner CV, five compounds are removed at a time (at random). The model parameter combination resulting in the lowest root mean squared error between the original and predicted binding affinities is selected and used in the model training in the outer CV loop. Note that also the rows and columns corresponding to the compounds included in the test fold are removed from the drug kernel matrix before model training. We used LDO-CV to tune the model parameters and assess its performance under the *New Drug* scenario (Fig 2b).
