# Supplementary material for: Computational-experimental approach to drug-target interaction mapping: A case study on kinase inhibitors
Source: PLoS Comput Biol. 2017 Aug 7;13(8):e1005678. doi: 10.1371/journal.pcbi.1005678 (PMC5560747; doi:10.1371/journal.pcbi.1005678)
Supplement: S14 Fig — Corresponding pIC50 values are summarized in S2 Table. (PDF) [file pcbi.1005678.s014.pdf]

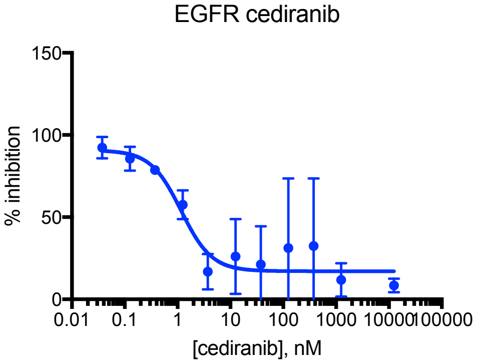
EGFR cediranib
% inhibition
150
100
50
0
0.01
0.1
1
10
100
1000
10000
100000
[cediranib], nM

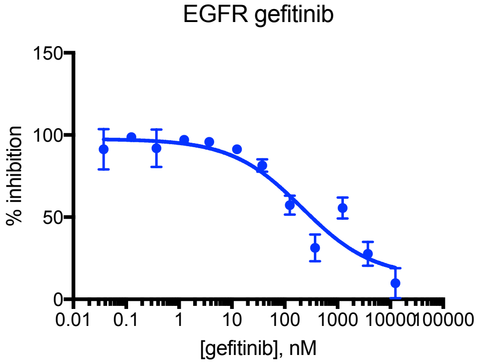
EGFR gefitinib
% inhibition
150
100
50
0
0.01
0.1
1
10
100
1000
10000
100000
[gefitinib], nM

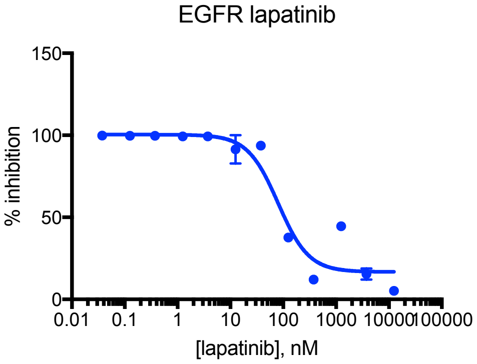
EGFR lapatinib
% inhibition
150
100
50
0
0.01
0.1
1
10
100
1000
10000
100000
[lapatinib], nM

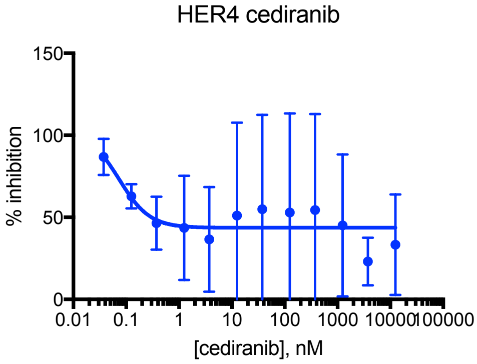
HER4 cediranib
% inhibition
150
100
50
0
0.01
0.1
1
10
100
1000
10000
100000
[cediranib], nM

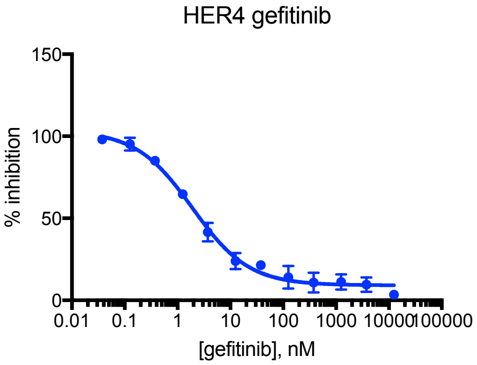
HER4 gefitinib
% inhibition
150
100
50
0
0.01
0.1
1
10
100
1000
10000
100000
[gefitinib], nM

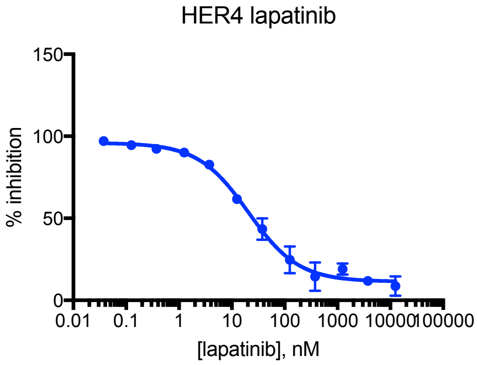
HER4 lapatinib
% inhibition
150
100
50
0
0.01
0.1
1
10
100
1000
10000
100000
[lapatinib], nM

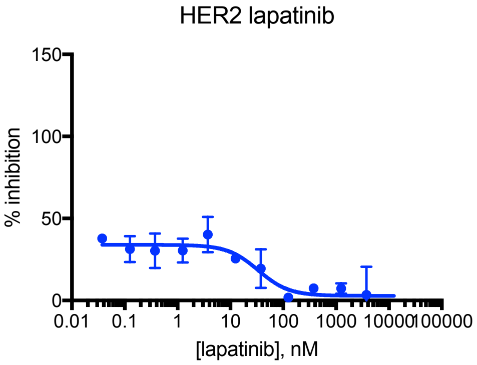
HER2 lapatinib
% inhibition
150
100
50
0
0.01
0.1
1
10
100
1000
10000
100000
[lapatinib], nM

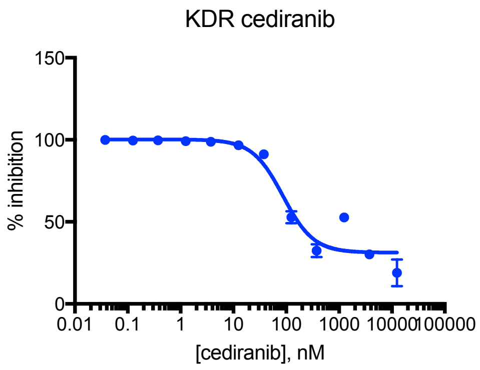
KDR cediranib
150
100
50
0
% inhibition
0.01
0.1
1
10
100
1000
10000
100000
[cediranib], nM

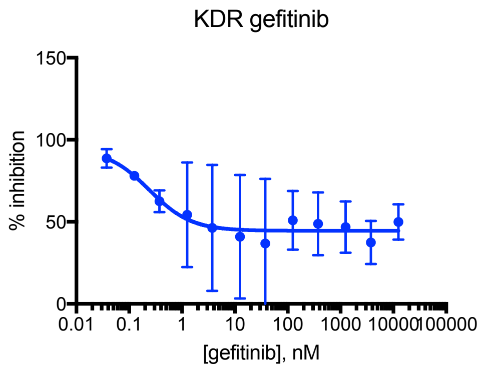
KDR gefitinib
150
100
50
0
% inhibition
0.01
0.1
1
10
100
1000
10000
100000
[gefitinib], nM

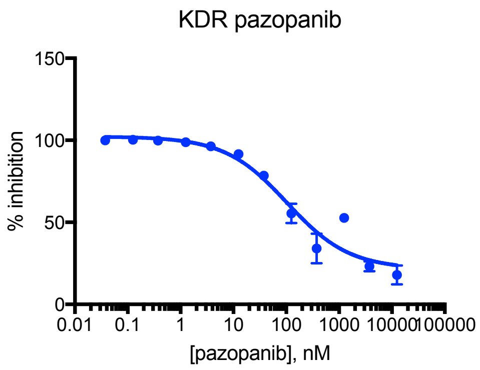
KDR pazopanib
150
100
50
0
% inhibition
0.01
0.1
1
10
100
1000
10000
100000
[pazopanib], nM

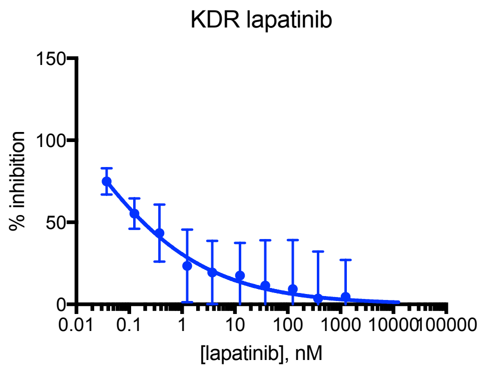
KDR lapatinib
150
100
50
0
% inhibition
0.01
0.1
1
10
100
1000
10000
100000
[lapatinib], nM

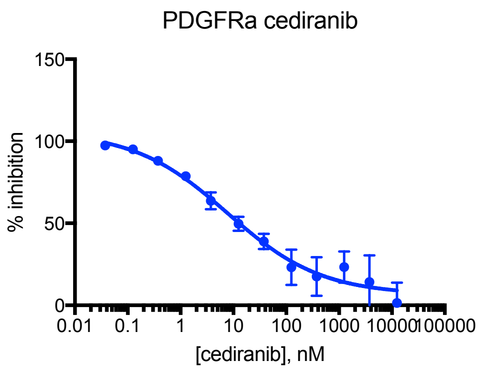
PDGFRa cediranib
150
100
50
0
% inhibition
0.01
0.1
1
10
100
1000
10000
100000
[cediranib], nM

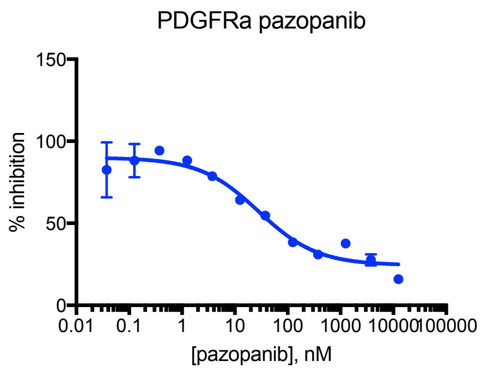
PDGFRa pazopanib
150
100
50
0
% inhibition
0.01
0.1
1
10
100
1000
10000
100000
[pazopanib], nM

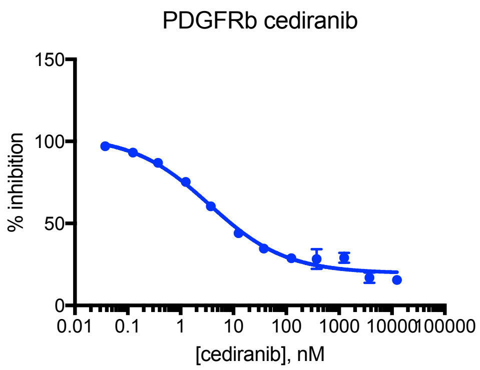
PDGFRb cediranib
150
100
50
0
% inhibition
0.01
0.1
1
10
100
1000
10000
100000
[cediranib], nM

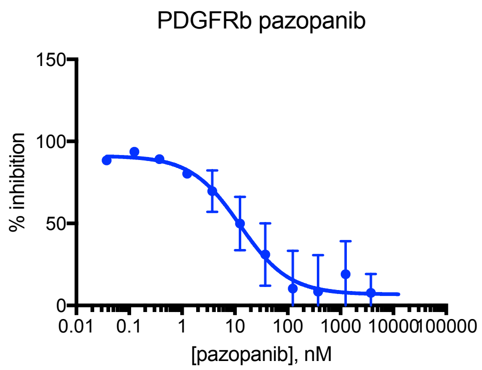
PDGFRb pazopanib
150
100
50
0
% inhibition
0.01
0.1
1
10
100
1000
10000
100000
[pazopanib], nM

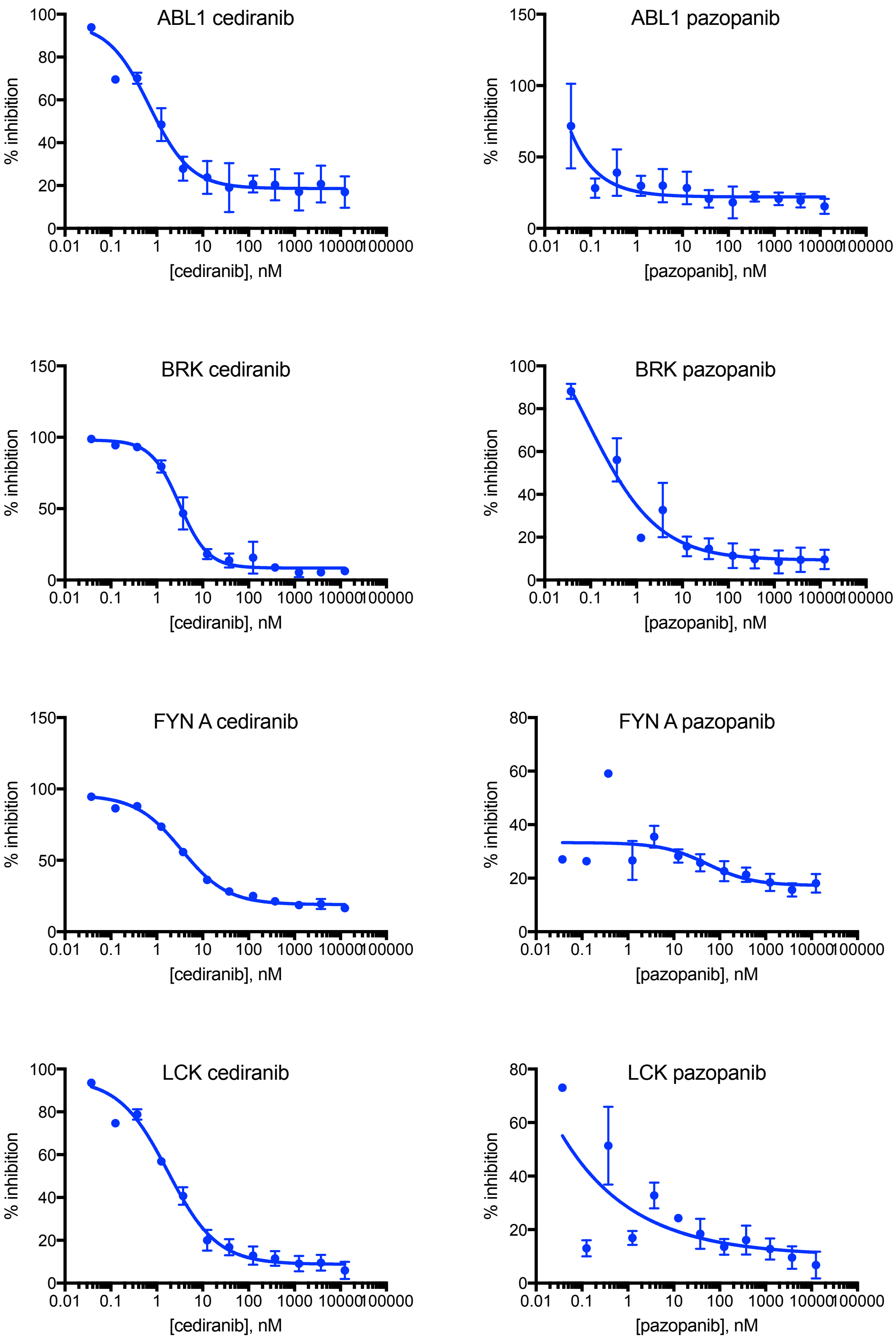
ABL1 cediranib
ABL1 pazopanib
BRK cediranib
BRK pazopanib
FYN A cediranib
FYN A pazopanib
LCK cediranib
LCK pazopanib
% inhibition
[cediranib], nM
[pazopanib], nM
0.01 0.1 1 10 100 1000 10000 100000
0 20 40 60 80 100
0 50 100 150

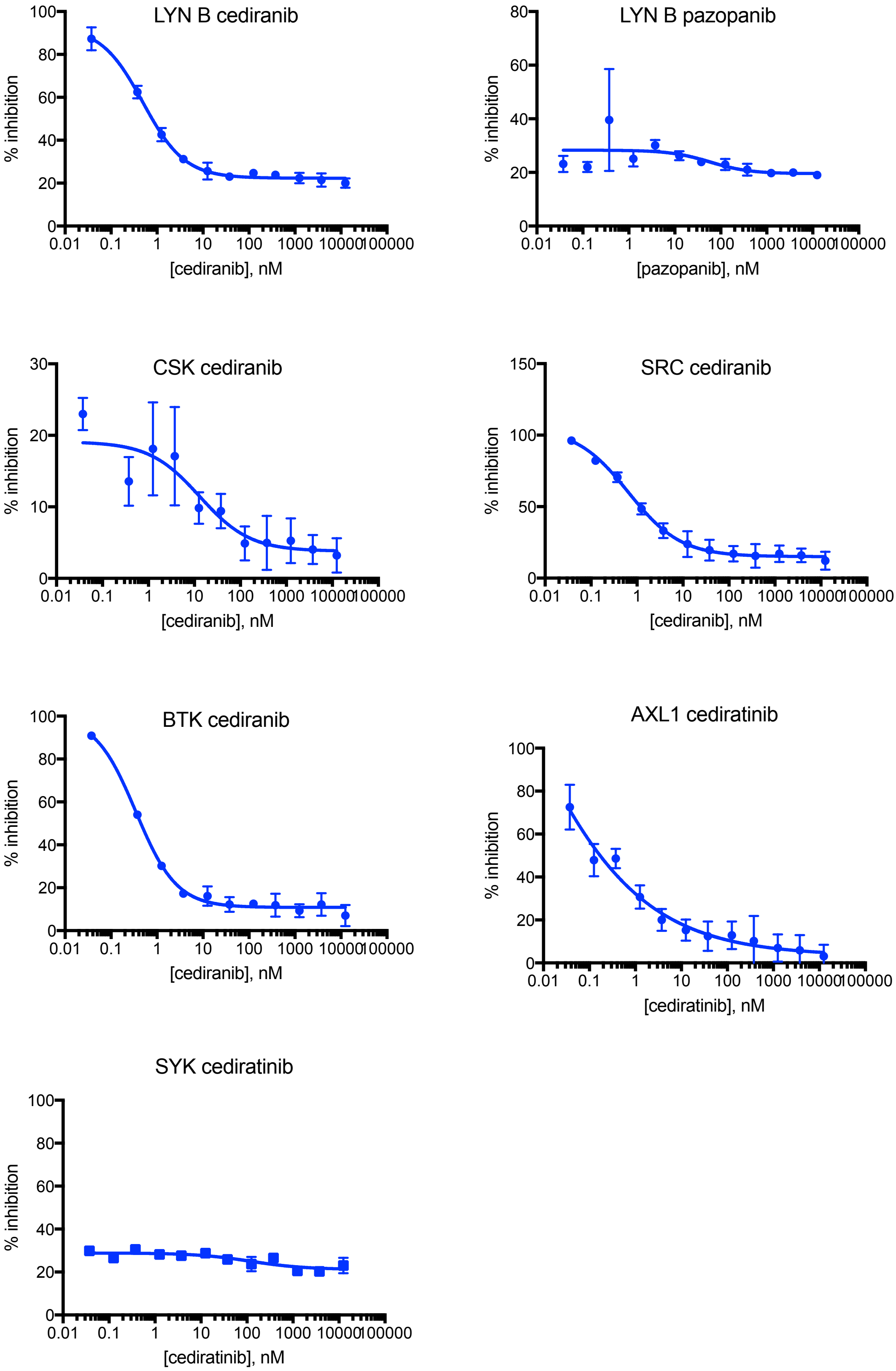

S14 Fig. Results of our kinase assay for testing bioactivities predicted to fill the experimental gaps in the large-scale kinase inhibitor target profiling study by Metz *et al.*; examples of drug response curves obtained as described in Materials and Methods section of the main paper. Corresponding pIC_{50} values are summarized in S2 Table.
