# Supplementary material for: Computational-experimental approach to drug-target interaction mapping: A case study on kinase inhibitors
Source: PLoS Comput Biol. 2017 Aug 7;13(8):e1005678. doi: 10.1371/journal.pcbi.1005678 (PMC5560747; doi:10.1371/journal.pcbi.1005678)
Supplement: S15 Fig — Corresponding pIC50 values are summarized in S3 Table. (PDF) [file pcbi.1005678.s015.pdf]

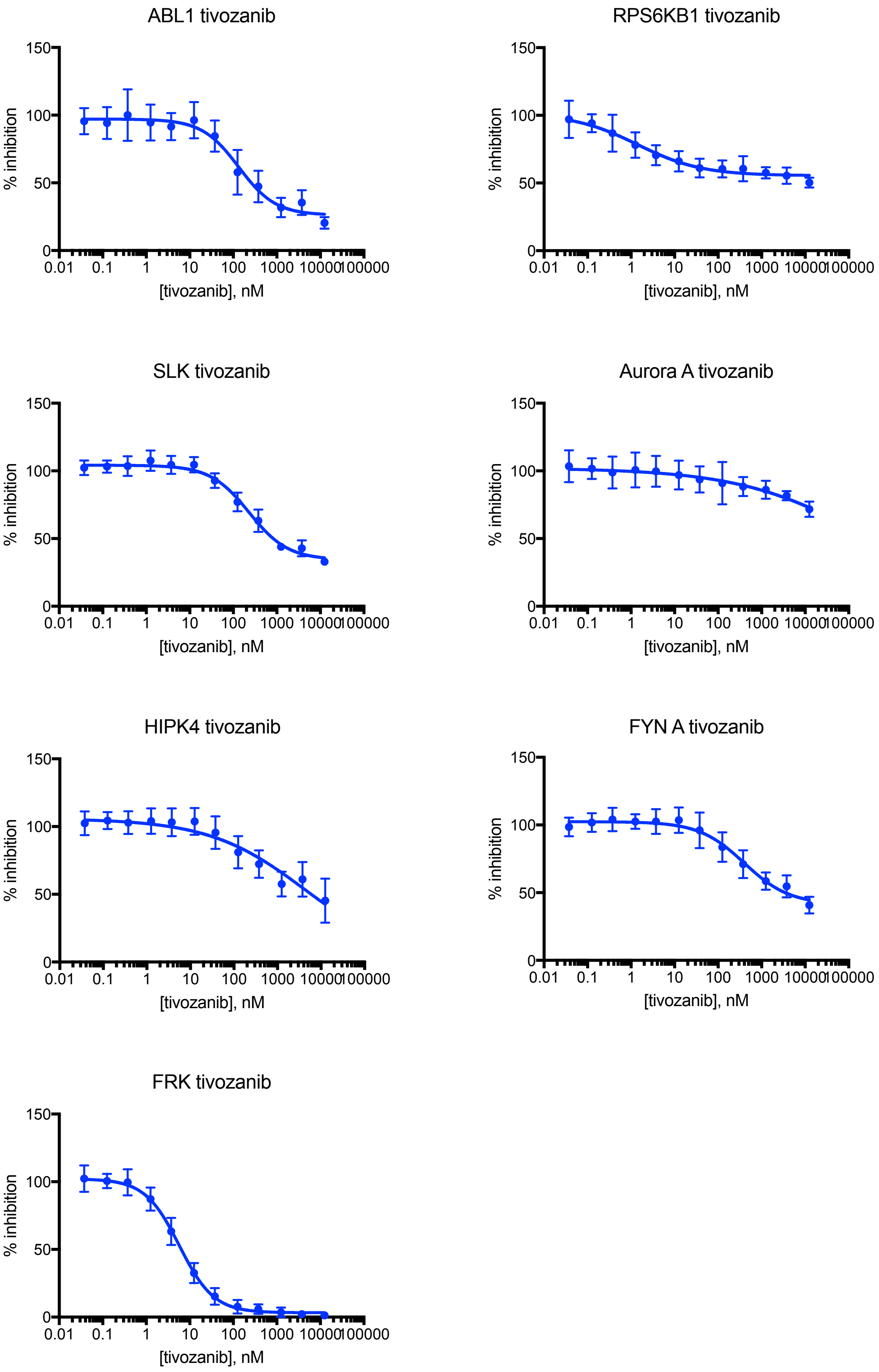

S15 Fig. Results of our kinase assay for testing predicted target interactions for a new investigational kinase inhibitor tivozanib; drug response curves obtained as described in Materials and Methods section of the main paper. Corresponding pIC_{50} values are summarized in S3 Table.
