# Supplementary material for: Computational-experimental approach to drug-target interaction mapping: A case study on kinase inhibitors
Source: PLoS Comput Biol. 2017 Aug 7;13(8):e1005678. doi: 10.1371/journal.pcbi.1005678 (PMC5560747; doi:10.1371/journal.pcbi.1005678)
Supplement: S1 Table — In this work, we focused on two most practical ones, namely the Bioactivity Imputation (Fig 2A) and the New Drug (Fig 2B) scenarios. Additionally, we included the results under the New Target setup (Fig 2C) in S7 Fig. (dx, px) denotes the query drug-protein pair, the binding affinity of which one aims to predict. (PDF) [file pcbi.1005678.s017.pdf]

S1 Table. Possible drug-protein interaction prediction scenarios. In this work, we focused on two most practical ones, namely the *Bioactivity Imputation* (Fig 2a) and the *New Drug* (Fig 2b) scenarios. Additionally, we included the results under the *New Target* setup (Fig 2c) in S7 Figure. (d_x, p_x) denotes the query drug-protein pair, the binding affinity of which one aims to predict.

Prediction scenario	Description of the application case	Cross-validation imitating the scenario
Bioactivity Imputation	Both the drug d_x and protein p_x are present in the training set (there exist known bioactivity values for the drug d_x and protein p_x, but not the pair (d_x, p_x), Fig 2a).<br>This scenario corresponds to having scattered missing values in otherwise known drug-protein interaction matrix. The aim is to predict the missing entries, including (d_x, p_x).	Leave-one-out cross-validation (LOO-CV).<br>Bioactivity of one particular drug-protein pair at a time is removed from the training data and kept as a test fold.
New Drug	The protein p_x is present in the training set, the drug d_x is not (there exist known bioactivity values for the protein p_x but not the drug d_x, Fig 2b).<br>It corresponds to the problem of predicting targets of an investigational drug compound which has no bioactivity data available for the model training.	Leave-drug-out cross-validation (LDO-CV).<br>All bioactivity values of one particular drug at a time are removed from the training data and kept as a test fold.
New Target	The drug d_x is present in the training set, the protein p_x is not (there exist known bioactivity values for the drug d_x, but not the protein p_x, Fig 2c).<br>It resembles the situation where the prediction is done for a protein which has no bioactivity data available for the model training.	Leave-target-out cross-validation (LTO-CV).<br>All bioactivity values of one particular protein at a time are removed from the training data and kept as a test fold.
New Drug-Target Pair	Neither the drug d_x nor the protein p_x is present in the training set (there exist no bioactivity values neither for the drug d_x nor the protein p_x, Fig 2d).<br>The aim is to predict a binding affinity between, for instance, a pair of a recently-discovered protein and an investigational drug compound.	Leave-drug-and-target-out cross-validation (LDTO-CV).<br>All bioactivity values of a particular drug and a particular protein at a time are removed from the training data and kept as a test fold.
