# Supplementary material for: Computational-experimental approach to drug-target interaction mapping: A case study on kinase inhibitors
Source: PLoS Comput Biol. 2017 Aug 7;13(8):e1005678. doi: 10.1371/journal.pcbi.1005678 (PMC5560747; doi:10.1371/journal.pcbi.1005678)
Supplement: S4 Table — In case of Gaussian kernels (KD-GIP, KP-GIP), the values for kernel width parameter σ were selected by computing pairwise distances between all data points and taking 0.1, 0.5 and 0.9 quantiles. (PDF) [file pcbi.1005678.s020.pdf]

S4 Table. Model parameters tested. In case of Gaussian kernels ($\mathbf{K}_{D\text{-}GIP}$, $\mathbf{K}_{P\text{-}GIP}$), the values for kernel width parameter σ were selected by computing pairwise distances between all data points and taking 0.1, 0.5 and 0.9 quantiles.

Parameter	Values tested
λ of KronRLS algorithm	10^{-5}, 10^{-4}, 10^{-3}, 10^{-2}, 10^{-1}, 10^{0}, 10^{1}, 10^{2}, 10^{3}, 10^{4}, 10^{5}
L of GS kernels $\mathbf{K}_{P\text{-}GS}$ and $\mathbf{K}_{P\text{-}GS\text{-}domain}$	5, 10, 20
L of GS kernel $\mathbf{K}_{P\text{-}GS\text{-}atp}$	5, 10
σ_p=σ_c of all GS kernels	1.0, 1.5, 2.0
σ of drug GIP kernel $\mathbf{K}_{D\text{-}GIP}$	4.8, 7.7, 11.6
σ of protein GIP kernel $\mathbf{K}_{P\text{-}GIP}$	5.3, 7.6, 11.3
