# Supplementary material for: Computational-experimental approach to drug-target interaction mapping: A case study on kinase inhibitors
Source: PLoS Comput Biol. 2017 Aug 7;13(8):e1005678. doi: 10.1371/journal.pcbi.1005678 (PMC5560747; doi:10.1371/journal.pcbi.1005678)
Supplement: S5 Table — (PDF) [file pcbi.1005678.s021.pdf]

S5 Table. Kinase inhibitors used in our experimental assays.

Kinase inhibitor	ChEMBL ID	CAS	Vendor	Product code
Cediranib	CHEMBL491473	288383-20-0	Selleck Chemicals	S1017
Lapatinib	CHEMBL554	388082-78-8	LC Laboratories	L-4804
Gefitinib	CHEMBL939	184475-35-2	LC Laboratories	G-4408
VX-745	CHEMBL119385	209410-46-8	Tocris Biosciences	3915
Pazopanib	CHEMBL477772	444731-52-6	LC Laboratories	P-6706
Tivozanib	CHEMBL1289494	475108-18-0	ChemieTek	CT-AV951
