# Supplementary material for: Computational-experimental approach to drug-target interaction mapping: A case study on kinase inhibitors
Source: PLoS Comput Biol. 2017 Aug 7;13(8):e1005678. doi: 10.1371/journal.pcbi.1005678 (PMC5560747; doi:10.1371/journal.pcbi.1005678)
Supplement: S6 Table — (PDF) [file pcbi.1005678.s022.pdf]

S6 Table. Kinases, substrates, and their concentrations used in tivozanib's off-target testing.

Kinase	Kinase final concentration (ng/µl)	Kinase product code	Substrate	Substrate final concentration (mg/mL)	Substrate product code
ABL1	1.5	A03-18H-10	Abltide synthetic peptide substrate (EAIYAAPFAKKK)	0.2	A02-58
RPS6KB1	10	R21-10H-10	S6K synthetic peptide substrate (CKRRRLASLR)	0.2	S05-58
SLK	20	S11-10G-10	Human histone H3 protein substrate	0.2	H12-54N
Aurora A	2.5	A28-18G-10	Myelin basic protein (MBP)	0.1	M42-51N
HIPK4	5	H06-10G-10	Myelin basic protein (MBP)	0.1	M42-51N
FYN A	2.5	F15-10G-10	Poly (Glu:Tyr, 4:1) synthetic peptide substrate	0.2	P61-58
FRK	2.5	F14-11G-10	Poly (Glu:Tyr, 4:1) synthetic peptide substrate	0.2	P61-58
