# Supplementary material for: Computational-experimental approach to drug-target interaction mapping: A case study on kinase inhibitors
Source: PLoS Comput Biol. 2017 Aug 7;13(8):e1005678. doi: 10.1371/journal.pcbi.1005678 (PMC5560747; doi:10.1371/journal.pcbi.1005678)
Supplement: S8 Fig — The bar length is proportional to the number of compound interactions for each kinase (pKi ≥ 7 M). On-targets of tivozanib are marked with red lines (FLT1, FLT4, KDR), fedratinib–green lines (JAK2, JAK3, TYK2), vx11e and ulixertinib (a compound derived from vx11e)–blue lines (vx11e: CDK2, ERK2; ulixertinib: ERK2). (PDF) [file pcbi.1005678.s008.pdf]

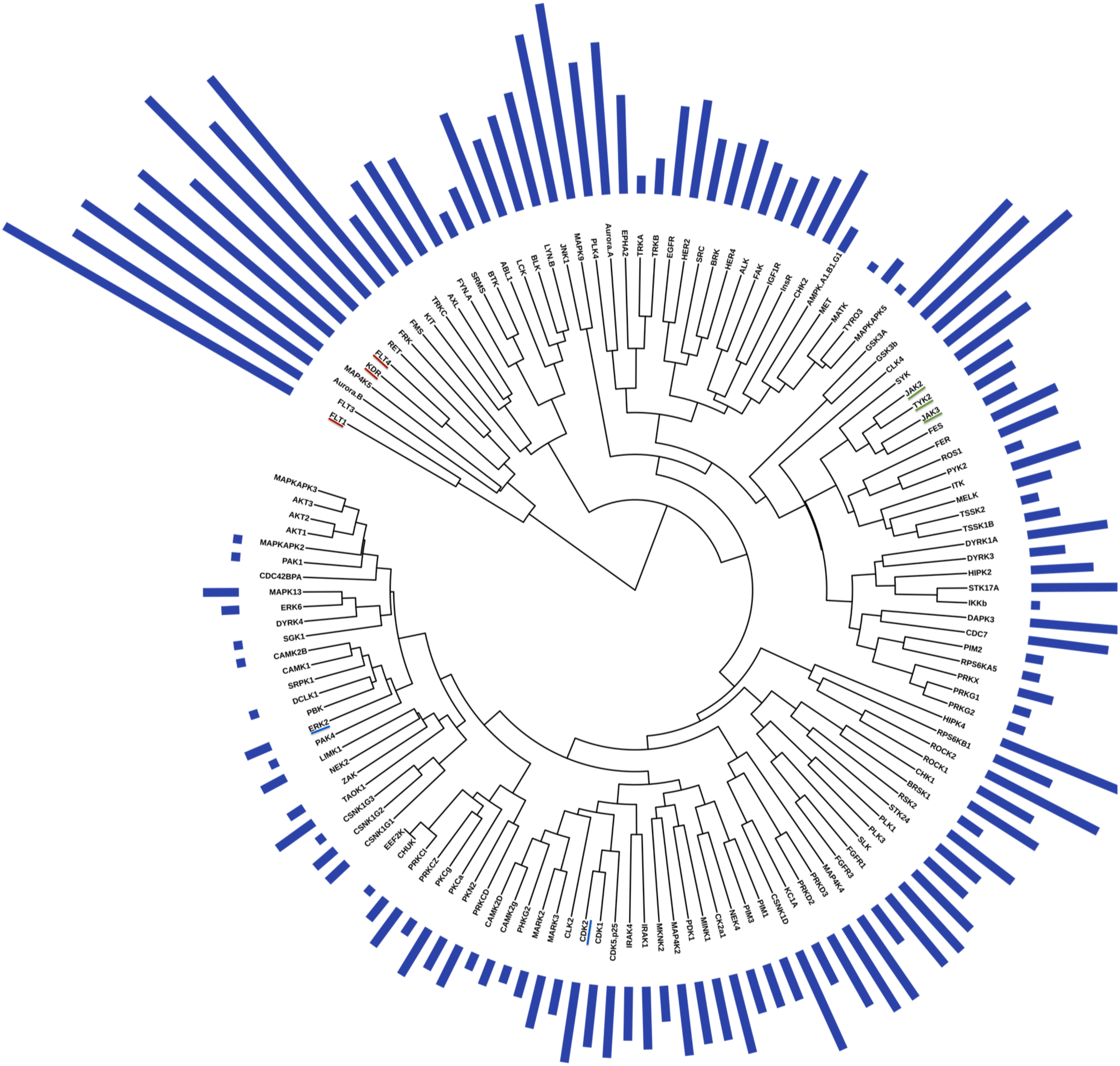

S8 Fig. Kinase dendrogram created by hierarchical clustering of 138 kinases from Metz *et al.* study based on their bioactivity data (pK_i). The bar length is proportional to the number of compound interactions for each kinase ($pK_i \geq 7$ M). On-targets of tivozanib are marked with red lines (*FLT1*, *FLT4*, *KDR*), fedratinib – green lines (*JAK2*, *JAK3*, *TYK2*), VX11e and ulixertinib (a compound derived from VX11e) – blue lines (VX11e: *CDK2*, *ERK2*; ulixertinib: *ERK2*).
